# Evaluation and improvement of isothermal amplification methods for point-of-need plant disease diagnostics

**DOI:** 10.1371/journal.pone.0235216

**Published:** 2020-06-29

**Authors:** Yiping Zou, Michael Glenn Mason, Jose Ramon Botella

**Affiliations:** School of Agriculture and Food Sciences, The University of Queensland, Brisbane, QLD, Australia; University of Helsinki, FINLAND

## Abstract

A number of isothermal DNA amplification technologies claim to be ideal for point-of-need (PON) applications as they enable reactions to be performed using a single-temperature heat source (e.g. water bath). Thus, we examined several isothermal amplification methods focusing on simplicity, cost, sensitivity and reproducibility to identify the most suitable method(s) for low resource PON applications. A number of methods were found unsuitable as they either involved multiple temperature incubations, were relatively expensive or required relatively large amounts target DNA for amplification. Among the methods examined, loop-mediated isothermal amplification (LAMP) and recombinase polymerase amplification (RPA) were found to be the most suitable for PON applications as they are both single step methods that provide highly sensitive and reproducible amplifications. The speed of LAMP reactions was greatly enhanced, up to 76%, with the addition of loop primers while the presence of swarm primers and the sequestration of free magnesium ions with nucleotides also enhanced the amplification speed. In contrast, we were unable to enhance RPA’s performance from the original published literature. While both RPA and LAMP have some drawbacks, either isothermal technology can reliably be used for on-site diagnostics with minimal equipment.

## Introduction

Disease diagnosis is the critical first step for disease management which is typically performed in modern laboratory facilities by skilled personnel due to the complexity of the techniques involved. Testing facilities are often located far away from sampling sites, which results in increased costs incurred by sample transportation as well as delays in disease identification and management. Thus, it would be highly beneficial to perform diagnostics at the point-of-need (PON), such as in a field-based environment [1], as it enables rapid implementation of disease management strategies [2–4].

DNA amplification is a powerful platform technology for diagnostics applications due to its speed, sensitivity, specificity, cost-effectiveness and adaptability [5, 6]. The most widely used amplification method is the polymerase chain reaction (PCR) [7] however, the requirement for a thermal cycler makes PCR-based diagnostics more suitable for laboratory-based testing than PON diagnostics [8]. The demand for PON molecular diagnostics has recently spurred significant interest in isothermal (single temperature) DNA amplification methods as they only require a simple, low-cost heat source [9]. Coupled with heat blocks, portable instruments or water baths, isothermal amplification has been used to detect causative disease agents in humans (e.g. *Campylobacter* [10], Chikungunya virus [11]) and plants (e.g. *Fusarium oxysporum* [12], flavescence dorée phytoplasma [13]).

DNA amplification at a constant temperature is typically made possible by the strand displacement ability of the DNA polymerases used in isothermal methods [6]. In most isothermal methods, the DNA polymerases enzymatically separate the DNA strands instead of relying on elevated temperatures as in PCR [14–17]. Other isothermal methods such as recombinase polymerase amplification (RPA) [18] utilize enzymes such as DNA recombinase to facilitate primer annealing while single stranded DNA binding proteins hold the DNA stands apart to allow the DNA polymerase to initiate DNA synthesis [19, 20]. Separation of the DNA strands during amplification can also be achieved using helicase as in helicase-dependent amplification (HDA) [21–24].

Isothermal DNA amplification has two key advantages over traditional PCR-based amplification for diagnostic applications. First, the device required to incubate the reaction can be significantly smaller and cheaper as it will have a lower power demand than a thermocycler and in some cases, it can be performed without electricity [25, 26][27]. Second, isothermal amplification is more sensitive and faster than PCR [28] as it does not rely on discrete thermal cycles like PCR, but rather involves continuous amplification that can result in detectable amplicons within 10 minutes. Furthermore, there are several reports that suggest that isothermal technologies such as LAMP with 6–8 primer binding sites can result in greater specificity than PCR-based methods [29, 30]. The combination of isothermal amplification with recent advances in diagnostic technologies such as the equipment-free dipstick nucleic acid purification technology [31] and naked eye-based readout technology [32], will facilitate the development of PON-capable molecular diagnostic platforms. In this study, we examined in detail several promising isothermal technologies to identify those most suitable for PON diagnostics. We evaluated the speed, cost, technical simplicity and detection limit of each method.

## Materials and methods

### Study design

The aim of this study was to identify suitable amplification method(s) for PON diagnostics. After initial assessment based on available information from the literature, several methods were selected due to their technical simplicity and relatively low cost and speed. These methods were then analyzed in greater detail, including detection limit, reproducibility, amplification speed and robustness, to identify suitable method(s) for PON purposes. In the last phase of this study, several modifications were made to two of these methods with the aim of improving either amplification speed or robustness.

### DNA template preparation

Purified DNA from *Fusarium oxysporum* f.sp. *conglutinans* and *Arabidopsis thaliana* ecotype Columbia were used as the target DNA and the non-target DNA, respectively, throughout this study.

Genomic DNA from *A*. *thaliana* was purified using a modified CTAB DNA extraction method [33]. *A*. *thaliana* leaves were ground into fine power using liquid nitrogen. Approximately 100 mg leaf power was added into 500 μl of 60°C extraction buffer (2% (w/v) CTAB, 1.42M NaCl, 20mM EDTA, 100mM Tris HCl [pH8.0], 1% w/v polyvinylyrilodone [PVP40]). The extract was incubated at 60°C for 45 minutes and then mixed with 500 μl of chilled chloroform: isoamyl alchohol (24:1, v/v). The mixture was gently rocked at room temperature for 15 minutes and then centrifuged at 15,000⊆g for 10 minutes. 200μl supernatant was transferred into a new tube and mixed with 400μl chilled ethanol before the mixture was centrifuged at 15,000⊆g for 10 minutes to pellet DNA. DNA pellet was washed with 80% ethanol, 100% ethanol successively. To denature RNA in the sample, the pellet was suspended with 100μl water and 50μg RNase A, and then incubated at 37 ^o^C for 20 minutes. The sample was mixed with 10μl of 3M sodium acetate and 100μl of isopropanol, followed by incubation at -20 ^o^C for 10 minutes. After centrifugation at 15,000g for 2 minutes, DNA pellet was obtained and then washed with 80% ethanol. After dry, DNA was suspended with 50μl water and quantified by NanoDrop ND-1000 spectrophotometer.

DNA from *F*. *conglutinans* was purified from pure liquid cultures. Briefly, three 5mm x 5mm agar blocks were excised from fresh culture plate and transferred into 200ml potato dextrose broth, and then incubated in the shaker at 28 ^o^C with the speed of 110 rpm. After approximately 4 days, the liquid culture was filtered through 4 layers of Miracloth to obtain *F*. *conglutinans* mycelium. The mycelium was then finely ground using liquid nitrogen. Approximately 100mg fine mycelium power was added into 400μl of extraction buffer (150mM Tris base, 2% w/v SDS, 50mM EDTA, 1% v/v b-mercaptoethanol, pH7.5) and vortexed for 5 minutes. 100μl of ethanol, 44μl of 5M potassium acetate were added into the extract respectively before 1-minute vortex upon addition of each reagent. 500μl chloroform: isoamyl alchohol (24:1, v/v) was added into mixture and vortexed for 1 minute, followed by centrifugation at maximum speed for 3 minutes. Approximately 500μl supernatant was transferred into a new tube and mixed with 500μl phenol: chloroform: isoamyl alcohol (25:24:1, v/v). After centrifugation at maximum speed for 3 minutes, 400 μl supernatant was mixed with 800μl chilled ethanol and then incubated at -20 ^o^C for 30 minutes. After centrifugation at 15,000⊆g for 10 minutes, the DNA was pelleted and then washed with 80% ethanol, followed by RNase treatment, ethanol wash, DNA quantification as described above in DNA extraction from *A*. *thaliana*.

### Primer design

Six LAMP primers sets (S1 Excel) were designed against the *endopolygalcturonase* gene of *F*. *oxysporum* (GeneBank: AB256753.1) according to the description detailed in the original LAMP publication [14] with the aid of PrimerExplorer v5 software (https://primerexplorer.jp/e/).

Three RPA primers sets, three CPA primers sets, eight PSR primers sets and three SEA primers sets (S1 Excel) were designed against *F*. *oxysporum endopolygalcturonase* gene as close to the LAMP primer position as possible according to the description detailed in its respective original publication [16–18, 34]. A modification to the CPA primer design was made by substituting primer 2a in original method with another primer named 6a that targeted a sequence downstream of the 5a primer (S3B Fig).

All primer sequences were checked for self- or cross-priming using ThermoFisher Scientific’s multiple Primer Analyzer (https://www.thermofisher.com/au/en/home/brands/thermo-scientific/molecular-biology/molecular-biology-learning-center/molecular-biology-resource-library/thermo-scientific-web-tools/multiple-primer-analyzer.html).

### Amplification

Unless otherwise stated, LAMP amplifications contained 0.8 M betaine, 8 mM MgSO_4_, 1.2 mM dNTP (each), 20 mM Tris-HCl (pH 8.8), 10 mM (NH_4_)_2_SO_4_, 10 mM KCl, 0.1% (v/v) Triton® X-100, 1.6 μM FIP primer, 1.6 μM BIP primer, 0.2 μM F3 primer, 0.2 μM B3 primer and 0.32 U/μl Bst 2.0 warm start polymerase (New England Biolab). 10 μl reactions were incubated at 63°C for 60 minutes and then at 85°C for 5 minutes for enzymes denaturation.

The components of CPA and PSR reactions were identical to LAMP reactions with the exception of the primers. 0.5 μM of 1s primer, 0.3 μM of 2a, 3a, 4s and 5a primers were used in CPA reactions, and 0.5 μM of 1s primer, 0.3 μM of 3a and 5a primers, and 0.05 μM of 4s and 6a primers were used in modified CPA reactions (mCPA). PSR reactions contained 1.6 μM of Ft and Bt, 0.8 μM of IF and IB. 10 μl reactions were incubated at 63°C for 60 minutes in CPA or 75 minutes in PSR followed by 85°C for 5 minutes.

SEA reactions contained 2 mM MgSO_4_, 0.5 mM dNTP(each), 20 mM Tris-HCl (pH 8.8), 10 mM (NH_4_)_2_SO_4_, 10 mM KCl, 0.1% Triton®-X-100, 10% (w/v) PEG-300, 1 μM of forward and reverse primers and 0.8 U/μl Bst 2.0 warm start polymerase. 10 μl reactions were incubated at 63°C for up to 180 minutes.

TwistAmp Basic RPA (Twist DX) was used as described in manufacturer’s instructions. Briefly, one RPA pellet was rehydrated with 29.5 μl of rehydration buffer, 0.48 μM of forward and reward primers and water are added to make up 42.5 μl solution. The mix was aliquoted evenly into five 0.2 ml tubes, and 1 μl DNA template was added. Unless otherwise stated, just prior to incubation 0.5 μl of 280 mM magnesium acetate was added into reactions. The 10 μl RPA reactions were then incubated at 37°C for 30 minutes, followed by 95°C for 5 minutes to denature proteins and release amplicon. After incubation, 2 μl of RPA products was mixed with 1 μl of dye containing 2% SDS and checked using agarose gel electrophoresis.

### Primer selection

Multiple sets of primers were designed for each isothermal method (S1 Excel) and tested using purified *F*. *conglutinans* DNA was used as the target DNA. Primer sets were excluded if they produced non-specific amplification in water control or did not produce any amplification from *F*. *conglutinans* DNA. One functional primer set (S1 Table) were selected for each method and used throughout remaining tests.

### Detection limit

0, 0.01, 0.1, 1, 5, 25 or 50 ng of purified *F*. *oxysporum* DNA was added into each type of isothermal reaction with final volume of 10 μl. Components in reactions and amplification conditions for each method were as described in the ‘Amplification’ section above.

### Real-time fluorescence assays

To monitor the reaction in real time, additional 1.25 μM SYTO9 (Invitrogen) was added to LAMP or RPA reactions. 1 ng purified *F*. *oxysporum* DNA and 1 ng *A*.*thaliana* DNA were added reactions as the target DNA and the non-target DNA, respectively. 10 μl reactions were monitored using Lightcycler 96 real time PCR machine (Roche) at respective reaction temperature with fluorescent measurements taken every 20 seconds.

From the raw real-time data, delta (Δ) fluorescence values were calculated by subtracting the starting fluorescence value of each sample from every value. To calculate the time for each sample to reach a set fluorescence threshold value (0.5 in LAMP and 0.05 in RPA), a minimum of 21 data points spanning the logarithmic growth phase were collected and fitted to a regression line with an r^2^ value of at least 0.95 in Microsoft Excel. TREND function was then used to calculate the time for each sample to reach the threshold value (T_threshold_).

### PCR amplification

10 μl PCR reactions contained 1 x FastStart essential DNA green master mix (Roche), 0.2 μM of each primer, and either 10 ng of target DNA, 10 ng of non-target DNA or water. Reactions were performed on Lightcycler 96 real-time thermocycler with the following parameters: 95°C for 10 minutes, followed by 45 cycles of 95°C for 10 seconds, 65°C for 10 seconds, 72°C for 20 seconds.

### Enhancement of LAMP amplification

To test whether RecA could accelerate LAMP, 0.75 μg RecA (New England Biolabs) and 1 mM ATP were added into 10 μl reactions (n = 10) containing either 0 M, 0.4 M or 0.8 M betaine. To investigate the effect of nucleotides in the absence of RecA, 0, 1, 2, 3, 4, 5, 6 or 7 mM ATP or 1 mM dATP, dTTP, dGTP or dCTP were added into 10 μl reactions containing 0.4 M betaine (n = 3) respectively. In further experiments, 4.8, 8.8, or 12.8 mM dNTPs (total) was added into 10 μl reactions containing 0.4 M betaine (n = 3) and either 8 mM or 10 mM Mg^2+^. To investigate effects of swarm and loop primers for LAMP amplification, the primers were designed (S1 Table) as described in their respective publications [35, 36]. 0.8 μM forward and reverse loop primers and/or 1.6 μM forward and reverse swarm primers were added into 10 μl reactions containing 0.4 M betaine (n = 4).

1 ng *F*. *oxysporum* DNA was used as the target DNA template. All reactions were performed on Lightcycler 96 at 63°C for 60 minutes followed by 85°C for 5 minutes.

### The effects of betaine on RPA function

Betaine was added into 10 μl of standard RPA reactions (described above, n = 4) containing 1 ng *F*. *oxysporum* DNA at a final concentration of 0, 0.4 or 0.8 M. All reactions were performed on Lightcyler 96 at 37°C for 60 minutes before denatured at 85°C for 5 minutes.

### Statistics

For comparison of two data sets, the data was analyzed using unpaired t-test (nonparametric) in GraphPad Prism (version 7.0b) (p ≤ 0.05). All other datasets were analyzed by performing one-way ANOVA followed by a post-hoc Tukey’s comparison of means test (p ≤ 0.05) in GraphPad Prism.

## Results

### Initial selection of isothermal amplification methods with potential PON applications

To identify the isothermal technologies that are suitable for PON applications, a large number of isothermal technologies were initially assessed in terms of technical simplicity, cost and speed based on the available literature. We excluded methods that involved multiple temperature incubations (e.g. strand displacement amplification [37]), were relatively expensive (e.g. helicase-dependent amplification [21]) or required special templates or multiple enzymatic reaction steps (e.g. rolling circle amplification [38]). From this assessment, we selected five methods for further work: strand exchange amplification (SEA), polymerase spiral reaction (PSR), cross priming amplification (CPA), recombinase polymerase amplification (RPA) and loop-mediated isothermal amplification (LAMP). SEA and RPA use forward and reverse primers similar to those employed in PCR, to amplify the target of interest (S1 Fig) and utilize a strand displacing DNA polymerase or a combination of recombinase and single stranded DNA binding proteins respectively to enable amplification at a single temperature. In contrast, LAMP, PSR and CPA use complex primer designs (S1 Fig) and strand displacing DNA polymerases to generate amplicons.

### Primer design and selection

To directly compare amplification methods, we designed multiple sets of primers against the same genomic region of the fungal plant pathogen *Fusarium oxysporum* f.sp. *conglutinans* (GenBank: AB256753.1, S1 Sequence) for each method (S1 Excel). All three RPA primer sets produced amplicons of the expected size for the target DNA (S2A Fig). Four of the six LAMP primers sets amplified the target DNA, while one set displayed non-specific amplification in water controls and the other failed to produce a product (S2B Fig). However, we had difficulty in finding reliable primer sets for the remaining three methods (CPA, SEA and PSR).

All eight PSR primer sets either failed to generate amplicons or displayed non-specific amplification in water controls when designed following the instructions from the original publication. However, follow-up publications described enhancements to PSR by adding additional inner primers (S1 Fig) [39, 40]. Implementation of this modification enabled successful amplification for one set of PSR primers (S2C Fig). Similarly, all three CPA primer sets, designed as described in the original publication [15], either failed to generate an amplicon from the target DNA or were prone to produce unspecific amplicons in the water controls (S3A Fig). However, amplification was achieved by keeping the same primer design as the original CPA method, that is, two forward and three reverse primers, but substituting the ‘2a’ primer with another primer (we named ‘6a’) further downstream (S3B Fig). We called this method modified CPA (mCPA) to distinguish it from the original published method. Our mCPA method displayed reliable amplifications from the target DNA and did not produce any amplification in water controls during testing (S3C Fig). Unfortunately, three primer sets designed for SEA amplification either failed to amplify from the target DNA or displayed non-specific amplification in water controls, and thus it was excluded from the remainder of our study. A single functional set of primers was selected for each of the remaining isothermal technologies and used throughout the rest of this study (S1 Table).

### Comparison of detection limits

**Fig 1:**
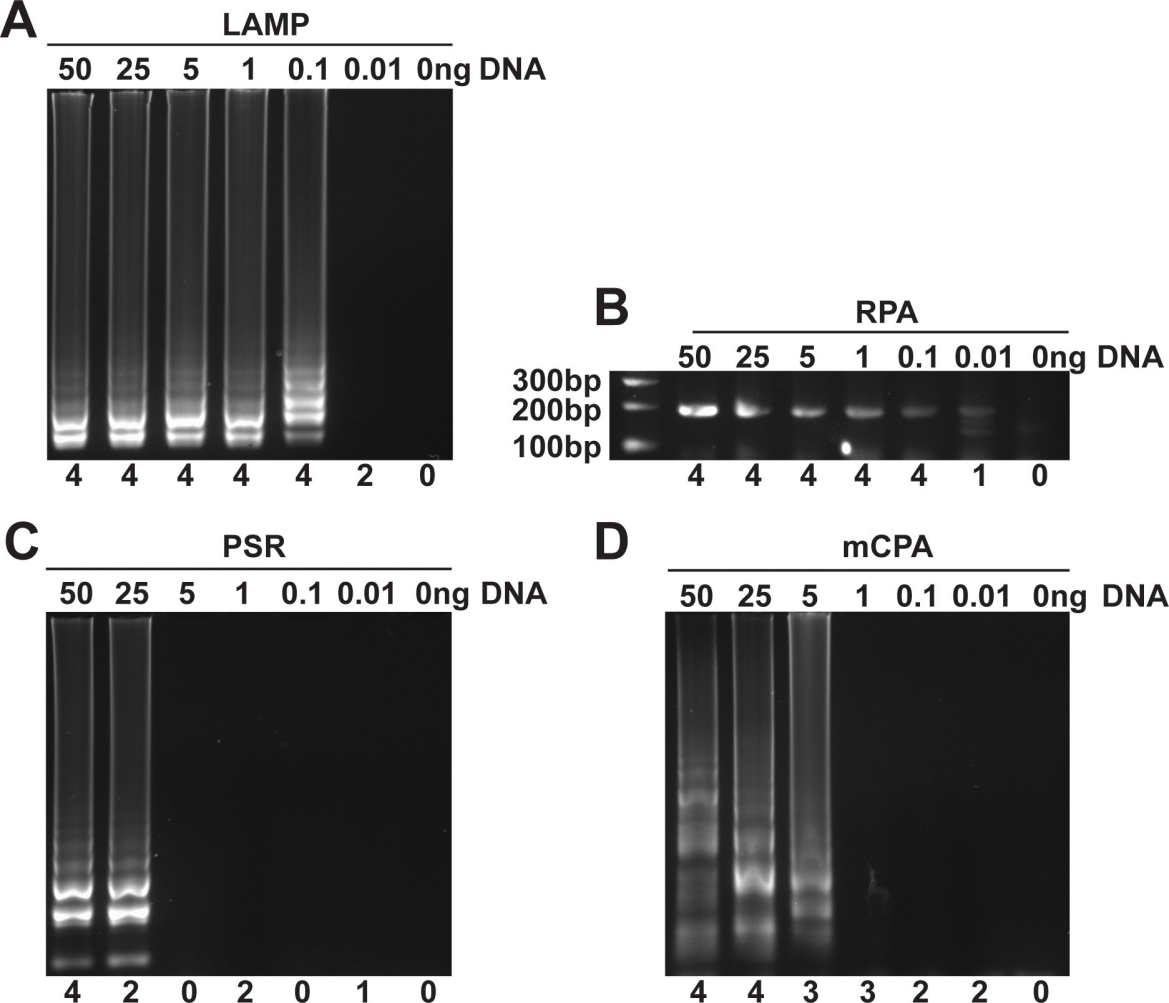
Detection limit of different isothermal amplification methods. Purified *F*. *oxysporum* genomic DNA (0, 0.01, 0.1, 1, 5, 25, 50 ng) were used as the target DNA for (**A**) LAMP, (**B**) RPA, (**C**) PSR or (**D**) mCPA amplification. The number below each lane shows how many times each method generated an amplicon from four independent experiments. Fig 1A and 1D originated from the same gel image, which is different from original gel image of either Fig 1B or Fig 1C (S1 Raw Images).

Next, we compared the sensitivity of each technology by testing their ability to produce an amplicon with different initial amounts (0.01 ng to 50 ng) of *F*. *oxysporum* genomic DNA in four independent experiments to assess the reproducibility of each method. LAMP produced visible amplicons in all four replicate reactions containing ≥ 0.1 ng DNA (Fig 1A), but only 50% of reactions produced an amplicon when 0.01 ng DNA was supplied. RPA produced almost identical results to LAMP with amplification in 100% of reactions with ≥ 0.1 ng DNA and 25% of reactions containing 0.01 ng DNA (Fig 1B). In contrast, both PSR and mCPA required higher amounts of DNA to reliably amplify a product. PSR showed 100% reproducibility when 50 ng of DNA was supplied, but did not consistently display amplification with lower template amounts (Fig 1C). mCPA generated amplicons in 100% of the reactions containing ≥ 25 ng, but did not display reproducible amplification with lower template amounts (Fig 1D). The relatively high amount of DNA needed for reproducible amplification in mCPA and PSR (250 and 500 times respectively higher than LAMP and RPA) makes them unsuitable for typical PON diagnostics [9] and were therefore excluded from further analysis.

### Comparison of amplification speed

**Fig 2:**
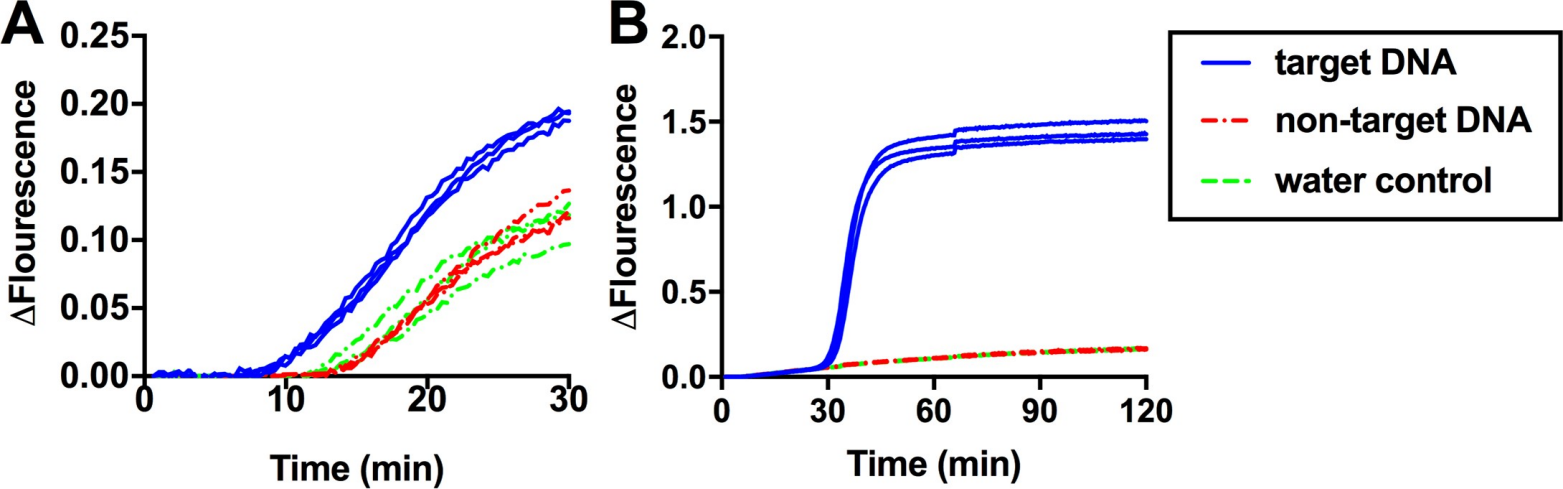
Real-time fluorescence detection of RPA and LAMP amplifications. 1ng *F*. *oxysporum* DNA was added into (**A**) RPA or (**B**) LAMP reactions (n = 3) as the target DNA (blue solid line). 1 ng *A*. *thaliana* genomic DNA and water were added into reactions (n = 3) as the non-target DNA (red dotted line) and water control (green dotted line) respectively.

We examined the time needed by LAMP and RPA to reliably distinguish between positive and negative reactions. Positive reactions contained purified *F*. *oxysporum* DNA (target DNA) while negatives contained either *A*. *thaliana* DNA (non-target DNA) or water. In RPA amplification, reactions containing the target DNA (n = 3) showed increases in fluorescence over background levels within 9 minutes from the start of the reaction. In contrast, water controls and non-target DNA controls displayed basal fluorescence levels after 14 minutes (Fig 2A). Consistent with the real-time results, analysis of RPA amplicons by gel electrophoresis revealed the presence of a strong, well defined DNA band of the expected size in positive samples. Importantly, all RPA reactions, including water and non-target DNA controls, produced non-specific amplicons (S4 Fig). Similar results were observed in three independent experiments in which new sets of RPA reagents were used. When the same RPA primers and templates were used in real-time PCR reactions using Taq polymerase, strong amplification signals were detected in the target DNA while no amplification occurred in either the non-target DNA and water controls (S5 Fig), indicating that the non-specific amplification products observed in RPA reactions are not caused by primer design or a contamination issue, but are inherent to RPA.

In LAMP reactions, the target DNA displayed amplification approximately 30 minutes after the start of incubation, while the non-target DNA and water controls showed basal fluorescence values during the 120-minute incubation (Fig 2B).

### Effect of preparation time on amplification

**Fig 3:**
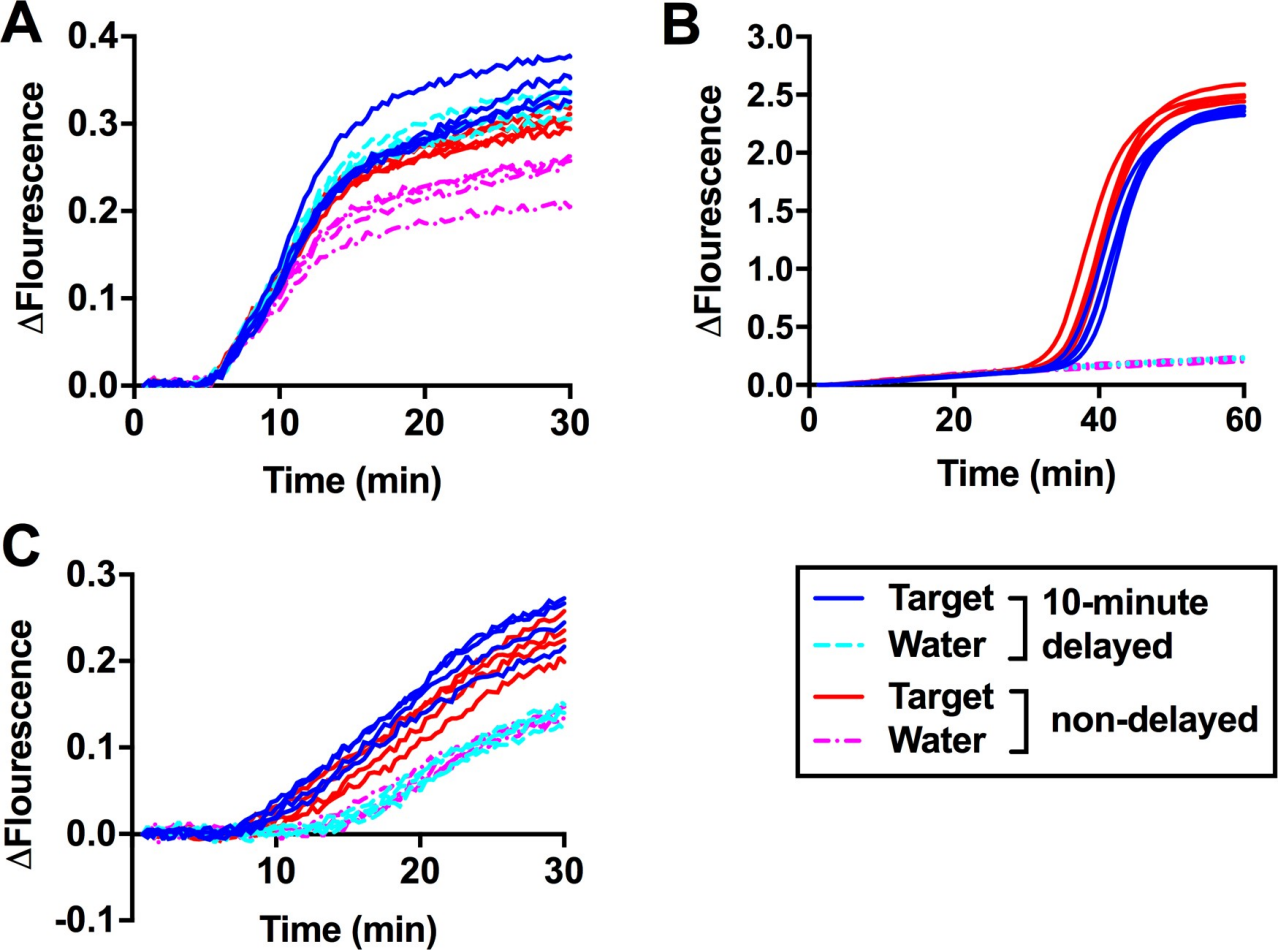
Effect of a 10-minute delay during sample preparation on RPA and LAMP amplifications. Two sets of (**A**) RPA or (**B**) LAMP complete reactions (n = 4) were prepared in advance. 1ng the target DNA (blue solid line) and water (cyan dotted line) were added into the first set of reactions 10 minutes prior to the start of incubation to simulate a 10-minute delay during sample preparation. Immediately prior to the start of incubation, target DNA (red solid line) or water (magenta dotted line) were added into the second set of reactions to simulate non-delayed samples. (**C**) Two sets of RPA reactions (n = 4) were prepared in the same way as in Fig 3A except that magnesium acetate was added to each sample immediately prior to the start of incubation.

In on-site situations, sample processing is not performed simultaneously especially in suboptimal weather or when numerous samples need to be processed, leading to delays. Thus, it is critical for diagnostic methods to tolerate small delays during sample preparation without affecting the reliability of the results. We purposely introduced a 10-minute delay between sample set up and incubation as this is in our experience the time required to process a small number (10–15) of reactions. Two identical sets of reactions were prepared in advance for both LAMP and RPA, containing all the required reagents except for the target DNA. The target DNA was added 10 minutes prior to the start of incubation to one set of reactions (n = 4), to simulate delayed samples, while for the second set (n = 4) the target DNA was added immediately before incubation to simulate non-delayed samples. Non-delayed RPA reactions showed similar results to our previous observations (Fig 2A), in which reactions containing the target DNA showed amplification slightly earlier than water controls (Fig 3A). Remarkably, delayed RPA reactions produced amplification curves in which target DNA and water controls were indistinguishable. In contrast, 10-minute delay did not affect the results of LAMP reactions (Fig 3B).

Next, we tested whether addition of magnesium acetate, a critical component in the RPA amplification reaction, immediately prior to the start of incubation could ameliorate the artifacts caused by the delay and restore the ability of RPA to distinguish between the target DNA and water controls. For this purpose, the target DNA was added to reactions (n = 4) lacking magnesium acetate either 10 minutes prior to or immediately before incubation at 37°C. Addition of magnesium acetate was performed immediately prior to incubation. Using this strategy, all water controls, delayed and not delayed, grouped together with the characteristic shift in amplification kinetics that allowed to differentiate them from the target DNA (Fig 3C).

### Enhancements to LAMP amplification

**Fig 4:**
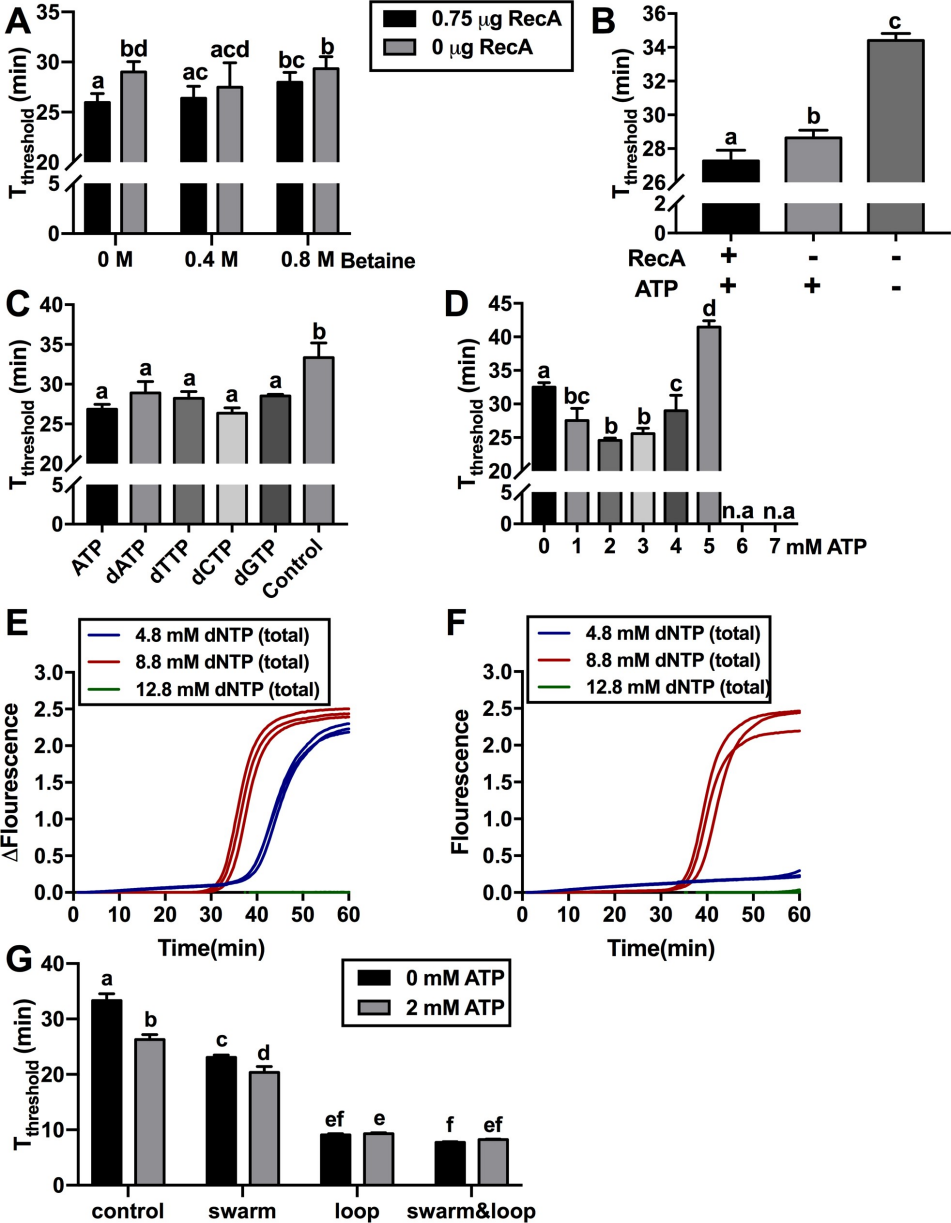
Effect of betaine, recombinase and nucleotide addition on LAMP amplification. (**A**) Different combinations of RecA and betaine were used in LAMP reactions containing 1 ng target DNA and 1 mM ATP and the time needed for each sample to reach the fluorescence threshold of 0.5 (T_threshold_) in real-time LAMP was determined (n = 10). (**B**) RecA and/or ATP were added into reactions containing 0.4 M betaine. Reactions without RecA and ATP were used as control (n = 3). “+” and “-” below each bar indicated the present and absent of the component respectively. (**C**) Additional 1 mM ATP, dATP, dTTP, dCTP or dGTP was added into reactions containing 0.4 M betaine. Reactions without additional ATP and dNTP were used as controls. (**D**) Different amounts of ATP were added into reactions containing 0.4 M betaine (n = 3). To investigate interplay between dNTPs and Mg^2+^, 4.8 mM (dark blue), 8.8 mM (red) or 12.8 mM (green) total dNTPs was added into reactions containing 0.4 M betaine and either (**E**) 8 mM Mg^2+^ or (**F**) 10 mM Mg^2+^ respectively. (**G**) Two sets of reactions containing 0.4 M betaine and either 0 mM (black) or 2 mM (grey) ATP were prepared. Additional swarm and/or loop primers along with standard LAMP primers were added into each set of reactions. Standard LAMP primers alone were used as controls (n = 4). All T_threshold_ data was analysed using one-way ANOVA with a post-hoc Tukey’s multiple comparison of means test (p value < 0.05). Error bars represent standard deviation.

A number of improvements to LAMP efficiency have been reported such as the addition of RecA recombinase along with its required ATP substrate [41] and the removal of betaine [42]. To evaluate the combined effect of both treatments, we compared the time needed for each sample to reach a fluorescence threshold of 0.5 in real-time LAMP, abbreviated as T_threshold_ in this study. The combination of RecA and 0 M betaine in the reaction resulted in a statistically significant reduction in T_threshold_ by three minutes compared to controls lacking RecA (n = 10, p value<0.05). Further analysis of the betaine-free LAMP reactions revealed that they were prone to produce false positives, with typically one out of every three reactions producing an amplicon in the absence of the target DNA, with or without the addition of RecA (S6A Fig). In contrast, reactions containing 0.8 M betaine did not produce any false positives (S6B Fig). As 0.4 M betaine, regardless of RecA addition, significantly decreased the T_threshold_ compared with standard LAMP reactions containing 0.8 M betaine and no RecA (Fig 4A), a minimum of 0.4 M betaine was used in all subsequent experiments.

Further experiments showed that the combination of RecA and ATP significantly reduced the T_threshold_ of LAMP reactions by seven minutes compared to reactions without these two components (Fig 4B). Surprisingly, we found that most of the improvement was due to the ATP since the addition of ATP alone, without RecA, reduced the T_threshold_ of the reaction by six minutes (Fig 4B).

Next, we tested whether LAMP reactions could be enhanced by nucleotides other than ATP. The T_threshold_ of reactions containing an additional 1 mM of dATP, dTTP, dCTP or dGTP were similar to those containing 1 mM ATP and significantly faster than control reactions without any additional nucleotides (Fig 4C). The optimal ATP concentration was 2–3 mM whereas ATP concentrations above 3 mM ATP had increasing detrimental effects and no amplification was observed with ATP concentrations of 6mM and above (Fig 4D).

To examine the interplay between magnesium and nucleotide concentration in LAMP reactions, we tested the effect of several dNTP and Mg^2+^ concentrations on amplification speed. When total dNTP concentration was increased from the standard 4.8 mM to 8.8 mM, T_threshold_ significantly decreased by 6 minutes (Fig 4E). However, further increasing total dNTP concentration to 12.8 mM completely inhibited amplification. When Mg^2+^ concentration was increased to 10 mM from the standard 8 mM, 4.8 mM dNTP was insufficient to enable amplification and a dNTP concentration of 8.8 mM was required for amplification (Fig 4F). Again, further increasing the dNTP concentration to 12.8 mM inhibited the reaction.

The addition of loop [35] and swarm primers [36] can increase the rate of LAMP amplification and thus, we examined whether the combination of ATP and these additional primers can further enhance amplification. In the absence of ATP, swarm or loop primers dramatically decreased T_threshold_ by ten and twenty-four minutes respectively (Fig 4G). In reactions containing loop primers, the addition of swarm primers did not further reduce the T_threshold_. The addition of ATP enhanced the rate of amplification in standard LAMP reactions and reactions containing swarm primers by seven and three minutes respectively, but did not reduce T_threshold_ in reactions containing loop primers.

**Fig 5:**
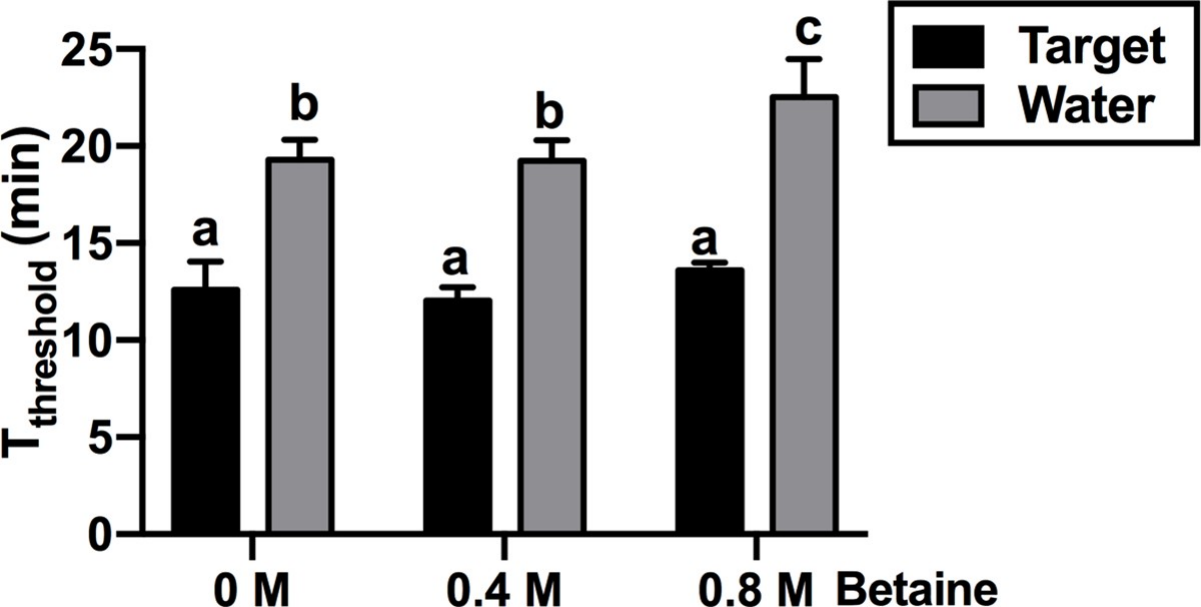
Effect of betaine on RPA amplification. 1ng target DNA (black) was added into RPA reactions containing different amount of betaine. Water (grey) was used as controls. The times needed for each sample to reach a fluorescence threshold of 0.05 (T_threshold_) in real-time RPA were analysed using one-way ANOVA (n = 4, p value<0.05). Error bars represent standard deviation.

Based on our observations that inclusion of betaine helped to avoid unspecific amplifications in LAMP reactions (S6 Fig), we tested whether betaine could also reduce or eliminate unspecific amplification in water controls of RPA. The addition of 0.4 or 0.8 M betaine did not significantly affect T_threshold_ in the presence of the target DNA compared to reactions lacking betaine (Fig 5). Non-specific amplification in water controls was not inhibited by the addition of 0.4 M betaine while the inclusion of 0.8 M betaine resulted in delayed production of non-specific amplicons by one minute.

## Discussion

In this study, we explored a number of isothermal amplification methods with potential for PON applications due to their reported simplicity, sensitivity and specificity. Our results revealed that, with the exception of RPA, primer design is more complex than it appears in the original publications. RPA primer design is similar to PCR and all RPA primer sets designed in this study successfully amplified their targets (S2A Fig). In contrast, 67% of the LAMP primer sets tested showed specific amplification of their target DNA (S2B Fig). This was not unexpected as LAMP requires a minimum of four primers to target six binding sites with strict requirements regarding the distances between each of the binding sites while each primer must also meet specific conditions such as fitting in a narrow range of melting temperatures [43]. The complicated LAMP primer design makes primer development time-consuming and suboptimal primers sets may have to be adopted when available genomic sequence is limited. However, in our experience, once a reliable set of primers has been developed, LAMP amplification is an easy-to-perform method with many advantages as a PON diagnostic tool. The recently developed SEA, CPA and PSR are simple one-step isothermal methods that utilize the same strand-displacing DNA polymerase as LAMP, but their primer designs are much simpler. However, none of the primer sets developed for the three methods by following the guidelines outlined in the original publications, were able to amplify their target DNA without non-specific amplification in water controls. The reasons for the poor primer performance for these isothermal methods are unknown but our results suggest that there are specific requirements for PSR, CPA and SEA that have not been documented. Subsequent modifications to PSR and CPA primer sets enabled specific amplification of the target DNA however, the sensitivity of the methods was several orders of magnitude lower compared to LAMP and RPA making them impractical for molecular diagnostic applications (Fig 1).

Our direct comparison of isothermal amplification methods showed that only LAMP and RPA provide suitable reliability and sensitivity for diagnostic purposes (Fig 1A and 1B) [9]. Compared to LAMP, RPA appears to be an attractive technology for diagnostics as it is both faster (Fig 2) and has a simpler primer design. However, unlike LAMP, RPA often produces non-specific amplification in water controls, which has been reported by other groups including the original inventors of the technology [18, 44, 45]. The non-specific amplification observed in RPA is not due to the primers used since the same primers did not result in unspecific amplicons when used in PCR reactions (S5 Fig). It is possible that when the reaction temperature is much lower than Tm values of primers, the recombinase used in RPA could mediate incorrect primer binding to off targets including trace amounts of *Escherichia coli* DNA, which is commonly co-purified with recombinant enzymes such as the polymerases and recombinases found in the RPA reaction [46, 47]. The addition of 0.8 M betaine to RPA reactions reduced the rate of non-specific amplification by 8% (Fig 5) but did not eliminate it as we observed in LAMP reactions (S6 Fig).

Gel electrophoresis can be used to differentiate target and non-specific RPA amplicons (S4 Fig) however, electrophoresis is not practical for PON applications as it requires a significant amount of laboratory equipment including a UV light box, a gel electrophoresis tank and associated power supply. A possible solution is to use specific probes to specifically detect the correct amplicons. The combination of labelled fluorescent probes [11, 48] and commercial portable real-time PCR machines is one viable option to enable specific RPA amplification at PON. Alternatively, the RPA reactions can be run on a lateral flow device (LFD) in which only the correct amplicon will be immobilized on the test band of the device and labelled with a specific probe [49, 50]. However, the LFD detection system adds considerable cost to the method and increases the risk of cross contamination of samples with previous RPA amplicons.

In contrast to RPA, the LAMP primer set selected for our work did not show any non-specific amplification during the 120-minute long incubation (Fig 2B) with the exception of the assays in which betaine was removed from the reaction (S6 Fig). Other research groups have also reported the absence of non-specific amplification in LAMP using long reaction times (90 to 150 minute) [51, 52]. The enhanced specificity of LAMP over RPA is likely a result of LAMP’s six primer binding sites and higher reaction temperatures that help to improve primer binding specificity [14].

We have confirmed that the timing of Mg^2+^ addition is critical for successful RPA amplification as delays during reaction set-up will result in similar amplification rates in the target and water control (Fig 3A and 3C). Magnesium is an essential co-factor for DNA polymerases [53, 54, 55] and thus the manufacturer of the RPA kits suggests adding it to the reaction just prior to incubation. Most components in RPA are supplied by the manufacturer in a freeze-dried pellet that allow to take components on site without refrigeration, which is ideal for PON applications. However, the requirement of pipetting a small volume of magnesium acetate to the RPA reaction immediately prior to incubation is not ideal for PON applications and, given the strict timing requirements of RPA, increases the risk of misinterpretation of the amplification result.

A major advantage of RPA over LAMP is its speed, thus, to enhance the rate of LAMP amplification, we investigated the effect of additional components on LAMP amplification speed. We found that the previously reported increase in the rate of LAMP amplification in the presence of RecA and ATP [41] was largely due to the presence of ATP rather than RecA (Fig 4B). Similar amplification improvements were also achieved using additional dNTPs; however, excessive amounts of ATP or dNTPs (>6mM) had a negative impact on the LAMP reaction (Fig 4D, 4E and 4F). This phenomenon is likely due to sequestration of free Mg^2+^ ions by an excess of negatively charged nucleotides (ATP and dNTPs). Magnesium is essential for DNA amplification as it is required for DNA polymerase function and it also reduces the repelling forces between the primers and the DNA template or between DNA strands due to their negatively charged phosphate backbone [56]. Thus, moderate sequestration of Mg^2+^ by dNTPs is likely to result in an increased number of localized DNA strand separation [57, 58], facilitating the initiation of LAMP amplification, while excessive nucleotide addition will reduce the levels of free Mg^2+^ to levels where DNA polymerase activity is inhibited. Consistent with this hypothesis, we found that when the amount of dNTP in a LAMP reaction is increased, additional magnesium was required for successful amplification (Fig 4E and 4F).

The rate of LAMP amplification can be further enhanced by the addition of loop primers [35] and swarm primers [36]. Addition of loop primers dramatically reduced the time needed for LAMP reactions to reach fluorescence threshold by 76% (from 33 to 8 minutes) (Fig 4G), close to the rate of RPA amplification (Fig 2A). However, the design of loop primers is not always possible since the target sequence may not have spaces accommodate loop primers. In addition, the presence of loop primers could result in the production of primer dimers. Although swarm primers were less efficient than loop primers (Fig 4G), design of swarm primers is much easier and generally do not produce primer dimer. Inclusion of ATP in reactions with swarm primers further accelerated amplification (Fig 4G), which could serve as a beneficial alternative option when loop primers cannot be developed.

The self-priming amplification system of LAMP results in vigorous DNA polymerase activity, which has been exploited for the development of a number of simple amplicon detection methods that are potentially suitable for PON applications. During LAMP amplification, a large amount of pyrophosphate ((P_2_O_7_)^4-^) is produced as a by-product of DNA amplification [34]. The combination of magnesium and pyrophosphate forms an insoluble magnesium pyrophosphate precipitate, which, as it accumulates, turns the reaction turbid. This increase in turbidity enables the user to visually or electronically determine whether or not an amplicon has been produced [31, 53, 54, 55]. Several colorimetric assays have also been developed for LAMP [31, 59–61] allowing the naked eye determination of a successful amplification. Like RPA, LAMP amplification can also be monitored using fluorescent dyes or labelled probes in conjunction with portable real time quantification devices [26, 62] which are suitable for PON applications.

## Conclusions

Our direct comparison of several isothermal amplification technologies revealed that LAMP and RPA are the best choices for PON applications. For RPA reactions, a specific target probe is required to distinguish between positive and negative samples due to the large amount of non-specific amplification. For LAMP, the complicated primer design is the major disadvantage, however, as we have demonstrated here, once a reliable set of primers have been developed, LAMP is comparable to RPA in terms of sensitivity and rate of amplification (when combined with loop primers) and does not produce non-specific amplification. The combination of these two isothermal technologies and their portable amplification readout methods with simple nucleic acid purification technologies [31, 63, 64] provides a practical means to perform on-site diagnostics and eliminate the need to transport samples to a laboratory for analysis. However, in low-resource environments, whereby measuring amplification by fluorescence is not possible/practical, LAMP is the ideal method to create robust, low-cost diagnostic assays.

## Supporting information

S1 Fig Primer designs of isothermal amplification technologies. Primers were simplified as lines with arrow heads indicating 5’ to 3’ direction. Primers indicated by green, blue and red lines target green, blue and red fragments of the template respectively. All other primers were aligned with their respective targeting regions. In SEA and RPA, one forward (F) and one reverse (R) primers are used. LAMP primers contain one forward (F3), one backward (B3) and two hybrid primers (FIP and BIP) targeting four different regions of the template. PSR primers contains two hybrid primers (Ft and Bt) with reverse sequence to each other at 5’ end and two inner primers (IF and IB) to enhance the amplification. CPA contains four normal primers (4s, 3a, 2a and 5a) and one hybrid primers (2s) with its sequence at 5’ end same with one normal primer (2a).
(TIF)

S2 Fig Primer identification. (**A**) RPA or (**B**) LAMP primers were added into its respective complete reactions to test the ability to amplify target DNA. (**C**) Additional PSR inner primers were added into PSR reactions that were indicated by ‘+’ sign. PSR reactions without inner primers were labelled by ‘-’ sign. Target, target DNA. Water, water control. Three panels in S2 Fig originated from three different raw gel images (S1 Raw Images).
(TIF)

S3 Fig The improvement on CPA primers. (**A**) Primers of original method were designed as described in original publication [15] and added into the master CPA reaction mix. 10 μl CPA reactions from the master mix were used to test the performance of the primers by adding either target DNA or water. (**B**) The diagram shows names and targeting regions of two sets of CPA primers either from original method or modified method. Each primer was simplified as one arrow with its direction indicating 5’-3’ direction. The 5’ end of primer 1s (red line) has the same sequence with primer 2a (red arrow) and targets the same region (red fragment in template) with 2a. Primers of modified method was obtained by substituting primer 2a with primer 6s. (**C**) Purified target DNA or water were added into 10 μl CPA reactions aliquot from the same master mix containing primers of modified method. Images of S3C Fig were generated from the same gel image, but re-arranged to remove irrelevant lanes. Panel (A) and (C) originated from different raw gel images.
(TIF)

S4 Fig Non-specific amplicons generated in RPA amplification. 1 ng *F*. *oxysporum* DNA was used as target DNA in reactions to produce 200 bp target amplicons. 1 ng *A*. *thaliana* DNA was used as non-target DNA. Reactions post amplification were analysed on agarose gel.
(TIF)

S5 Fig RPA primers tested by real-time PCR. RPA primers were added into PCR reactions containing either 10 ng target DNA (blue solid line), non-target DNA (red dotted line) or water (green dotted line).
(TIFF)

S6 Fig False amplification of water controls in betaine-free LAMP. reactions. Water was added into reactions containing betaine and/or RecA. 1 ng *F*. *oxysporum* (target DNA) was added into the reaction without betaine and RecA as positive control. Images in each panel were generated from the same raw gel image, but re-arranged to remove irrelevant lanes in respective gel images (S1 Raw Images).
(TIF)

S1 Table Prominent primers used in this study. (DOCX)

S1 Sequence 209 bp fragment from *F*.*conglutinans* (GenBank: AB256753.1) for primer design. (DOCX)

S1 Excel Oligonucleotide sequences. (XLSX)

S1 Raw Images (PDF)

## References

[1] Donoso A, Valenzuela S. In‐field molecular diagnosis of plant pathogens: recent trends and future perspectives. Plant Pathol. 2018.

[2] Clerc O, Greub G. Routine use of point-of-care tests: usefulness and application in clinical microbiology. Clin Microbiol Infect. 2010;16(8):1054–61. 10.1111/j.1469-0691.2010.03281.x 20670287

[3] Yager P, Domingo GJ, Gerdes J. Point-of-care diagnostics for global health. Annu Rev Biomed Eng. 2008;10. 10.1146/annurev.bioeng.10.061807.160524 18358075

[4] Peeling R, Mabey D. Point-of-care tests for diagnosing infections in the developing world. Clin Microbiol Infect. 2010;16(8):1062–9. 10.1111/j.1469-0691.2010.03279.x 20670288

[5] Ward E, Foster SJ, Fraaije BA, Mccartney HA. Plant pathogen diagnostics: immunological and nucleic acid‐based approaches. Ann Appl Biol. 2004;145(1):1–16.

[6] Craw P, Balachandran W. Isothermal nucleic acid amplification technologies for point-of-care diagnostics: a critical review. Lab Chip. 2012;12(14):2469–86. 10.1039/c2lc40100b 22592150

[7] Mullis K, Faloona F, Scharf S, Saiki R, Horn G, Erlich H, editors. Specific enzymatic amplification of DNA in vitro: the polymerase chain reaction Cold Spring Harbor symposia on quantitative biology; 1986: Cold Spring Harbor Laboratory Press. 10.1101/sqb.1986.051.01.032 3472723

[8] Zhao Y, Chen F, Li Q, Wang L, Fan C. Isothermal amplification of nucleic acids. Chem Rev. 2015;115(22):12491–545. 10.1021/acs.chemrev.5b00428 26551336

[9] Niemz A, Ferguson TM, Boyle DS. Point-of-care nucleic acid testing for infectious diseases. Trends Biotechnol. 2011;29(5):240–50. 10.1016/j.tibtech.2011.01.007 21377748 PMC3746968

[10] Mason MG, Blackall PJ, Botella JR, Templeton JM. An easy‐to‐perform, culture‐free Campylobacter point‐of‐management assay for processing plant applications. J Appl Microbiol. 2019. 10.1111/jam.14509 PMC7027919 31705613

[11] Patel P, El Wahed AA, Faye O, Prüger P, Kaiser M, Thaloengsok S, et al A field-deployable reverse transcription recombinase polymerase amplification assay for rapid detection of the chikungunya virus. PLoS Neglected Trop Dis. 2016;10(9):e0004953. 10.1371/journal.pntd.0004953 PMC5042537 27685649

[12] Lau HY, Wang Y, Wee EJ, Botella JR, Trau M. Field Demonstration of a Multiplexed Point-of-Care Diagnostic Platform for Plant Pathogens. Anal Chem. 2016;88(16):8074–81. 10.1021/acs.analchem.6b01551 27403651

[13] Kogovšek P, Hodgetts J, Hall J, Prezelj N, Nikolić P, Mehle N, et al LAMP assay and rapid sample preparation method for on‐site detection of flavescence dorée phytoplasma in grapevine. Plant Pathol. 2015;64(2):286–96. 10.1111/ppa.12266 26146413 PMC4480326

[14] Notomi T, Okayama H, Masubuchi H, Yonekawa T, Watanabe K, Amino N, et al Loop-mediated isothermal amplification of DNA. Nucleic Acids Res. 2000;28(12):e63–e. 10.1093/nar/28.12.e63 10871386 PMC102748

[15] Xu G, Hu L, Zhong H, Wang H, Yusa S-i, Weiss TC, et al Cross priming amplification: mechanism and optimization for isothermal DNA amplification. Sci Rep. 2012;2:246 10.1038/srep00246 22355758 PMC3271364

[16] Liu W, Dong D, Yang Z, Zou D, Chen Z, Yuan J, et al Polymerase Spiral Reaction (PSR): A novel isothermal nucleic acid amplification method. Sci Rep. 2015;5. 10.1038/srep12723 PMC4518254 26220251

[17] Shi C, Shang F, Zhou M, Zhang P, Wang Y, Ma C. Triggered isothermal PCR by denaturation bubble-mediated strand exchange amplification. Chem Commun. 2016;52(77):11551–4. 10.1039/c6cc05906f 27602549

[18] Piepenburg O, Williams CH, Stemple DL, Armes NA. DNA detection using recombination proteins. PLoS Biol. 2006;4(7):e204 10.1371/journal.pbio.0040204 16756388 PMC1475771

[19] Yonesaki T, Ryo Y, Minagawa T, Takahashi H. Purification and some of the functions of the products of bacteriophage T4 recombination genes, uvs X and uvs Y. Eur J Biochem. 1985;148(1):127–34. 10.1111/j.1432-1033.1985.tb08816.x 3156738

[20] Formosa T, Alberts B. Purification and characterization of the T4 bacteriophage uvsX protein. J Biol Chem. 1986;261(13):6107–18. 2939071

[21] Vincent M, Xu Y, Kong H. Helicase‐dependent isothermal DNA amplification. EMBO Rep. 2004;5(8):795–800. 10.1038/sj.embor.7400200 15247927 PMC1249482

[22] Runyon G, Lohman T. Escherichia coli helicase II (uvrD) protein can completely unwind fully duplex linear and nicked circular DNA. J Biol Chem. 1989;264(29):17502–12. 2529260

[23] Kornberg A, Baker TA. DNA replication: Wh Freeman New York; 1992.

[24] Caruthers JM, McKay DB. Helicase structure and mechanism. Curr Opin Struct Biol. 2002;12(1):123–33. 10.1016/s0959-440x(02)00298-1 11839499

[25] Singleton J, Osborn JL, Lillis L, Hawkins K, Guelig D, Price W, et al Electricity-free amplification and detection for molecular point-of-care diagnosis of HIV-1. PloS one. 2014;9(11):e113693 10.1371/journal.pone.0113693 25426953 PMC4245218

[26] Liao S-C, Peng J, Mauk MG, Awasthi S, Song J, Friedman H, et al Smart cup: a minimally-instrumented, smartphone-based point-of-care molecular diagnostic device. Sens Actuators, B. 2016;229:232–8. 10.1016/j.snb.2016.01.073 PMC4756427 26900258

[27] Nkouawa A, Sako Y, Li T, Chen X, Nakao M, Yanagida T, et al A loop-mediated isothermal amplification method for a differential identification of Taenia tapeworms from human: application to a field survey. Parasitol Int. 2012;61(4):723–5. 10.1016/j.parint.2012.06.001 22698671

[28] Zanoli LM, Spoto G. Isothermal amplification methods for the detection of nucleic acids in microfluidic devices. Biosensors. 2013;3(1):18–43. 10.3390/bios3010018 25587397 PMC4263587

[29] Khan M, Wang R, Li B, Liu P, Weng Q, Chen Q. Comparative evaluation of the LAMP assay and PCR-based assays for the rapid detection of Alternaria solani. Front Microbiol. 2018;9:2089 10.3389/fmicb.2018.02089 30233554 PMC6129767

[30] Wang X, Seo DJ, Lee MH, Choi C. Comparison of conventional PCR, multiplex PCR, and loop-mediated isothermal amplification assays for rapid detection of Arcobacter species. J Clin Microbiol. 2014;52(2):557–63. 10.1128/JCM.02883-13 24478488 PMC3911361

[31] Zou Y, Mason MG, Wang Y, Wee E, Turni C, Blackall PJ, et al Nucleic acid purification from plants, animals and microbes in under 30 seconds. PLoS Biol. 2017;15(11):e2003916 10.1371/journal.pbio.2003916 29161268 PMC5697807

[32] Mason MG, Botella JR. A simple, robust and equipment-free DNA amplification readout in less than 30 seconds. RSC Adv. 2019;9(42):24440–50. 10.1039/c9ra04725e PMC9069613 35527854

[33] Allen G, Flores-Vergara M, Krasynanski S, Kumar S, Thompson W. A modified protocol for rapid DNA isolation from plant tissues using cetyltrimethylammonium bromide. Nat Protoc. 2006;1(5):2320 10.1038/nprot.2006.384 17406474

[34] Mori Y, Nagamine K, Tomita N, Notomi T. Detection of loop-mediated isothermal amplification reaction by turbidity derived from magnesium pyrophosphate formation. Biochem Biophys Res Commun. 2001;289(1):150–4. 10.1006/bbrc.2001.5921 11708792

[35] Nagamine K, Hase T, Notomi T. Accelerated reaction by loop-mediated isothermal amplification using loop primers. Mol Cell Probes. 2002;16(3):223–9. 10.1006/mcpr.2002.0415 12144774

[36] Martineau RL, Murray SA, Ci S, Gao W, Chao S-h, Meldrum DR. Improved performance of loop-mediated isothermal amplification assays via Swarm priming. Anal Chem. 2016;89(1):625–32. 10.1021/acs.analchem.6b02578 27809497

[37] Walker GT, Fraiser MS, Schram JL, Little MC, Nadeau JG, Malinowski DP. Strand displacement amplification—an isothermal, in vitro DNA amplification technique. Nucleic Acids Res. 1992;20(7):1691–6. 10.1093/nar/20.7.1691 1579461 PMC312258

[38] Fire A, Xu S-Q. Rolling replication of short DNA circles. Proc Natl Acad Sci. 1995;92(10):4641–5. 10.1073/pnas.92.10.4641 7753856 PMC42000

[39] Dong D, Zou D, Liu H, Yang Z, Huang S, Liu N, et al Rapid detection of Pseudomonas aeruginosa targeting the toxA gene in intensive care unit patients from Beijing, China. Front Microbiol. 2015;6. 10.3389/fmicb.2015.01100 PMC4594016 26500639

[40] Jiang X, Dong D, Bian L, Zou D, He X, Ao D, et al Rapid Detection of Candida albicans by polymerase spiral reaction assay in clinical blood samples. Front Microbiol. 2016;7. 10.3389/fmicb.2016.00916 PMC4905949 27379048

[41] Mitsunaga S, Shimizu S, Okudaira Y, Oka A, Tanaka M, Kimura M, et al Improved loop-mediated isothermal amplification for HLA-DRB1 genotyping using RecA and a restriction enzyme for enhanced amplification specificity. Immunogenetics. 2013;65(6):405–15. 10.1007/s00251-013-0690-0 23474534

[42] Ma C, Wang Y, Zhang P, Shi C. Accelerated isothermal nucleic acid amplification in betaine-free reaction. Anal Biochem. 2017;530:1–4. 10.1016/j.ab.2017.04.017 28457896

[43] Owczarzy R, Tataurov AV, Wu Y, Manthey JA, McQuisten KA, Almabrazi HG, et al IDT SciTools: a suite for analysis and design of nucleic acid oligomers. Nucleic Acids Res. 2008;36(suppl_2):W163–W9. 18440976 10.1093/nar/gkn198 PMC2447751

[44] Aebischer A, Wernike K, Hoffmann B, Beer M. Rapid genome detection of Schmallenberg virus and bovine viral diarrhea virus by use of isothermal amplification methods and high-speed real-time reverse transcriptase PCR. J Clin Microbiol. 2014;52(6):1883–92. 10.1128/JCM.00167-14 24648561 PMC4042763

[45] Kim N-Y, Oh J, Lee S-H, Kim H, Moon JS, Jeong R-D. Rapid and specific detection of apple stem grooving virus by reverse transcription-recombinase polymerase amplification. The plant pathology journal. 2018;34(6):575 10.5423/PPJ.NT.06.2018.0108 30588230 PMC6305176

[46] Iulia L, Bianca IM, Cornelia O, Octavian P. The evidence of contaminant bacterial DNA in several commercial Taq polymerases. Biotechnol Lett. 2013;18:8007–12.

[47] Wilson IG. Inhibition and facilitation of nucleic acid amplification. Appl Environ Microbiol. 1997;63(10):3741 9327537 10.1128/aem.63.10.3741-3751.1997 PMC168683

[48] Wand NIV, Bonney LC, Watson RJ, Graham V, Hewson R. Point-of-care diagnostic assay for the detection of Zika virus using the recombinase polymerase amplification method. The Journal of general virology. 2018;99(8):1012 10.1099/jgv.0.001083 29897329 PMC6171711

[49] Rosser A, Rollinson D, Forrest M, Webster B. Isothermal Recombinase Polymerase amplification (RPA) of Schistosoma haematobium DNA and oligochromatographic lateral flow detection. Parasites Vectors. 2015;8(1):446. 26338510 10.1186/s13071-015-1055-3 PMC4559068

[50] Ahmed FA, Larrea-Sarmiento A, Alvarez AM, Arif M. Genome-informed diagnostics for specific and rapid detection of Pectobacterium species using recombinase polymerase amplification coupled with a lateral flow device. Sci Rep. 2018;8(1):1–11. 10.1038/s41598-017-17765-5 30374117 PMC6206099

[51] Scheel CM, Zhou Y, Theodoro RC, Abrams B, Balajee SA, Litvintseva AP. Development of a loop-mediated isothermal amplification method for detection of Histoplasma capsulatum DNA in clinical samples. J Clin Microbiol. 2014;52(2):483–8. 10.1128/JCM.02739-13 24478477 PMC3911334

[52] da Silva Gonçalves D, Cassimiro APA, de Oliveira CD, Rodrigues NB, Moreira LA. Wolbachia detection in insects through LAMP: loop mediated isothermal amplification. Parasites Vectors. 2014;7(1):228. 24885509 10.1186/1756-3305-7-228 PMC4033683

[53] Goto M, Honda E, Ogura A, Nomoto A, Hanaki K. Colorimetric detection of loop-mediated isothermal amplification reaction by using hydroxy naphthol blue. Biotechniques. 2009;46(3):167–72. 10.2144/000113072 . 19317660

[54] Tomita N, Mori Y, Kanda H, Notomi T. Loop-mediated isothermal amplification (LAMP) of gene sequences and simple visual detection of products. Nat Protoc. 2008;3(5):877 10.1038/nprot.2008.57 18451795

[55] Wee E, Lau H, Botella J, Trau M. Re-purposing bridging flocculation for on-site, rapid, qualitative DNA detection in resource-poor settings. Chem Commun. 2015;51(27):5828–31. 10.1039/c4cc10068a 25622026

[56] Markoulatos P, Siafakas N, Moncany M. Multiplex polymerase chain reaction: a practical approach. J Clin Lab Anal. 2002;16(1):47–51. 10.1002/jcla.2058 11835531 PMC6808141

[57] Altan-Bonnet G, Libchaber A, Krichevsky O. Bubble dynamics in double-stranded DNA. Phys Rev Lett. 2003;90(13):138101 10.1103/PhysRevLett.90.138101 12689326

[58] Jiang Y, Li B, Milligan JN, Bhadra S, Ellington AD. Real-time detection of isothermal amplification reactions with thermostable catalytic hairpin assembly. J Am Chem Soc. 2013;135(20):7430–3. 10.1021/ja4023978 23647466 PMC3724415

[59] Miyamoto S, Sano S, Takahashi K, Jikihara T. Method for colorimetric detection of double-stranded nucleic acid using leuco triphenylmethane dyes. Anal Biochem. 2015;473:28–33. 10.1016/j.ab.2014.12.016 25575759

[60] Tanner NA, Zhang Y, Evans TC Jr. Visual detection of isothermal nucleic acid amplification using pH-sensitive dyes. Biotechniques. 2015;58(2):59–68. 10.2144/000114253 25652028

[61] Hill J, Beriwal S, Chandra I, Paul VK, Kapil A, Singh T, et al Loop-mediated isothermal amplification assay for rapid detection of common strains of Escherichia coli. J Clin Microbiol. 2008;46(8):2800–4. 10.1128/JCM.00152-08 18550738 PMC2519505

[62] Liu C, Geva E, Mauk M, Qiu X, Abrams WR, Malamud D, et al An isothermal amplification reactor with an integrated isolation membrane for point-of-care detection of infectious diseases. Analyst. 2011;136(10):2069–76. 10.1039/c1an00007a 21455542 PMC4360993

[63] Tomlinson J, Dickinson M, Hobden E, Robinson S, Giltrap P, Boonham N. A five-minute DNA extraction method for expedited detection of Phytophthora ramorum following prescreening using Phytophthora spp. lateral flow devices. J Microbiol Methods. 2010;81(2):116–20. 10.1016/j.mimet.2010.02.006 20171248

[64] McFall SM, Wagner RL, Jangam SR, Yamada DH, Hardie D, Kelso DM. A simple and rapid DNA extraction method from whole blood for highly sensitive detection and quantitation of HIV-1 proviral DNA by real-time PCR. J Virol Methods. 2015;214:37–42. 10.1016/j.jviromet.2015.01.005 25681524

