# Supplementary figures and images for: Evaluation and improvement of isothermal amplification methods for point-of-need plant disease diagnostics
Source: PLoS One. 2020 Jun 29;15(6):e0235216. doi: 10.1371/journal.pone.0235216 (PMC7323990; doi:10.1371/journal.pone.0235216)

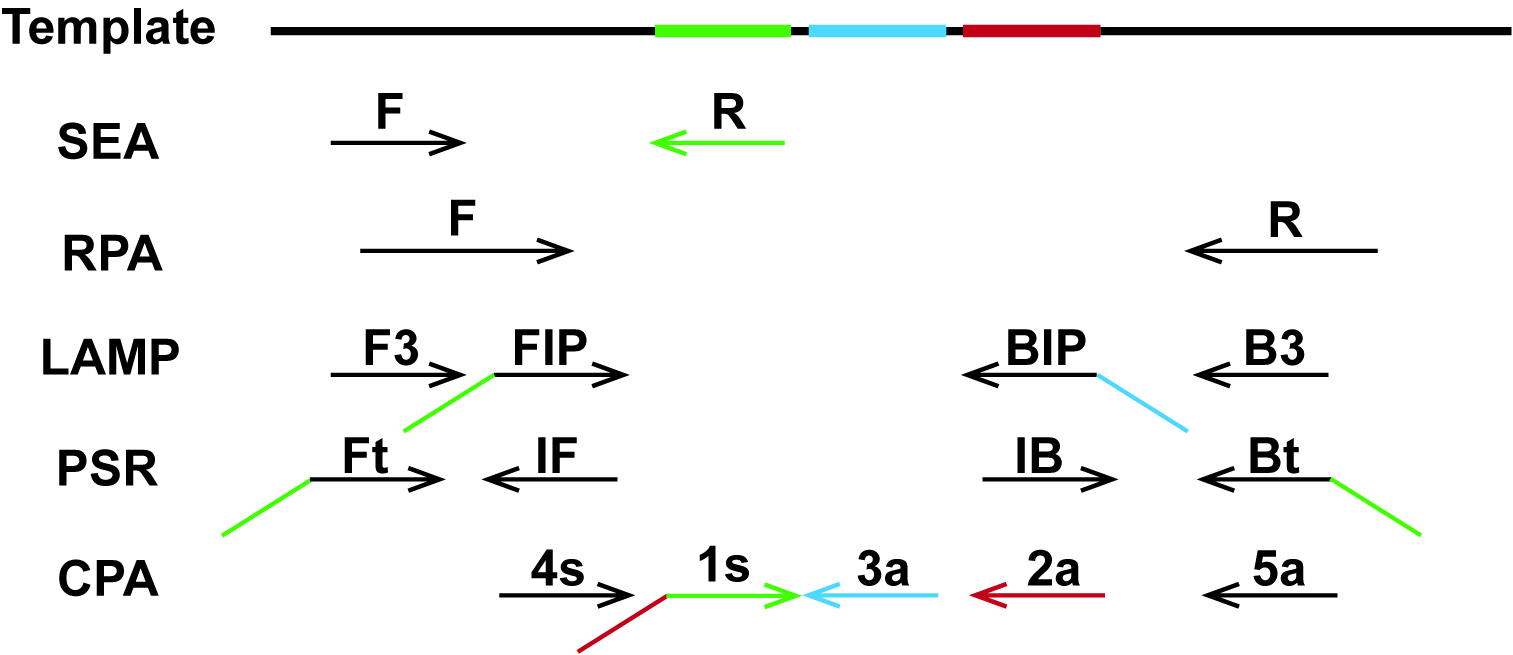

Supplement: S1 Fig — Primers were simplified as lines with arrow heads indicating 5’ to 3’ direction. Primers indicated by green, blue and red lines target green, blue and red fragments of the template respectively. All other primers were aligned with their respective targeting regions. In SEA and RPA, one forward (F) and one reverse (R) primers are used. LAMP primers contain one forward (F3), one backward (B3) and two hybrid primers (FIP and BIP) targeting four different regions of the template. PSR primers contains two hybrid primers (Ft and Bt) with reverse sequence to each other at 5’ end and two inner primers (IF and IB) to enhance the amplification. CPA contains four normal primers (4s, 3a, 2a and 5a) and one hybrid primers (2s) with its sequence at 5’ end same with one normal primer (2a). (TIF) [file pone.0235216.s001.tif]

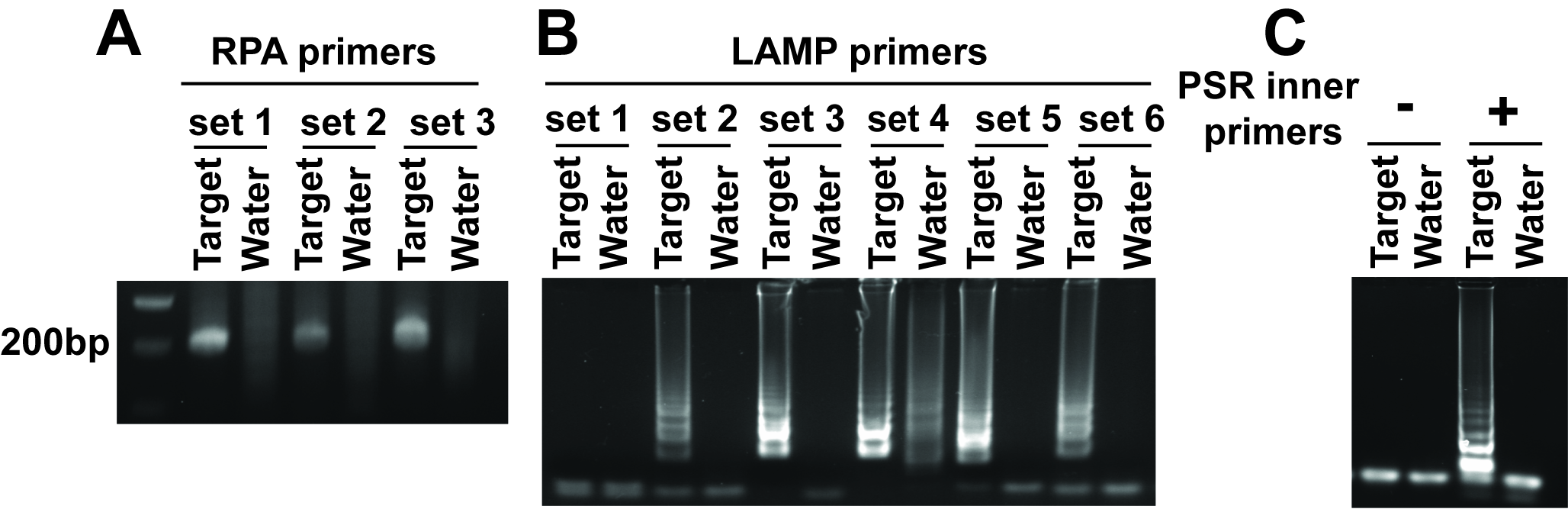

Supplement: S2 Fig — (A) RPA or (B) LAMP primers were added into its respective complete reactions to test the ability to amplify target DNA. (C) Additional PSR inner primers were added into PSR reactions that were indicated by ‘+’ sign. PSR reactions without inner primers were labelled by ‘-’ sign. Target, target DNA. Water, water control. Three panels in S2 Fig originated from three different raw gel images (S1 Raw Images). (TIF) [file pone.0235216.s002.tif]

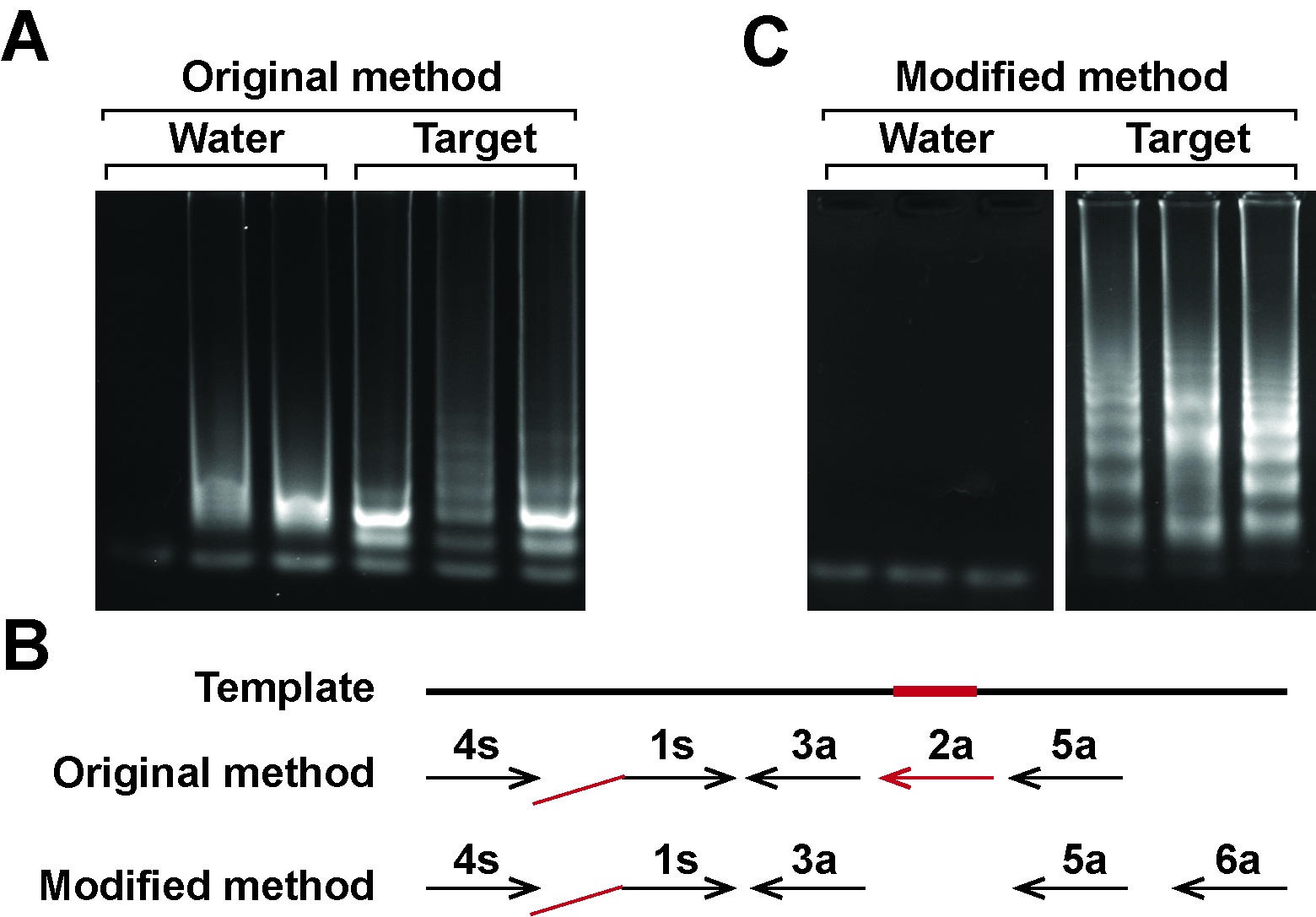

Supplement: S3 Fig — (A) Primers of original method were designed as described in original publication [15] and added into the master CPA reaction mix. 10 μl CPA reactions from the master mix were used to test the performance of the primers by adding either target DNA or water. (B) The diagram shows names and targeting regions of two sets of CPA primers either from original method or modified method. Each primer was simplified as one arrow with its direction indicating 5’-3’ direction. The 5’ end of primer 1s (red line) has the same sequence with primer 2a (red arrow) and targets the same region (red fragment in template) with 2a. Primers of modified method was obtained by substituting primer 2a with primer 6s. (C) Purified target DNA or water were added into 10 μl CPA reactions aliquot from the same master mix containing primers of modified method. Images of S3C Fig were generated from the same gel image, but re-arranged to remove irrelevant lanes. Panel (A) and (C) originated from different raw gel images. (TIF) [file pone.0235216.s003.tif]

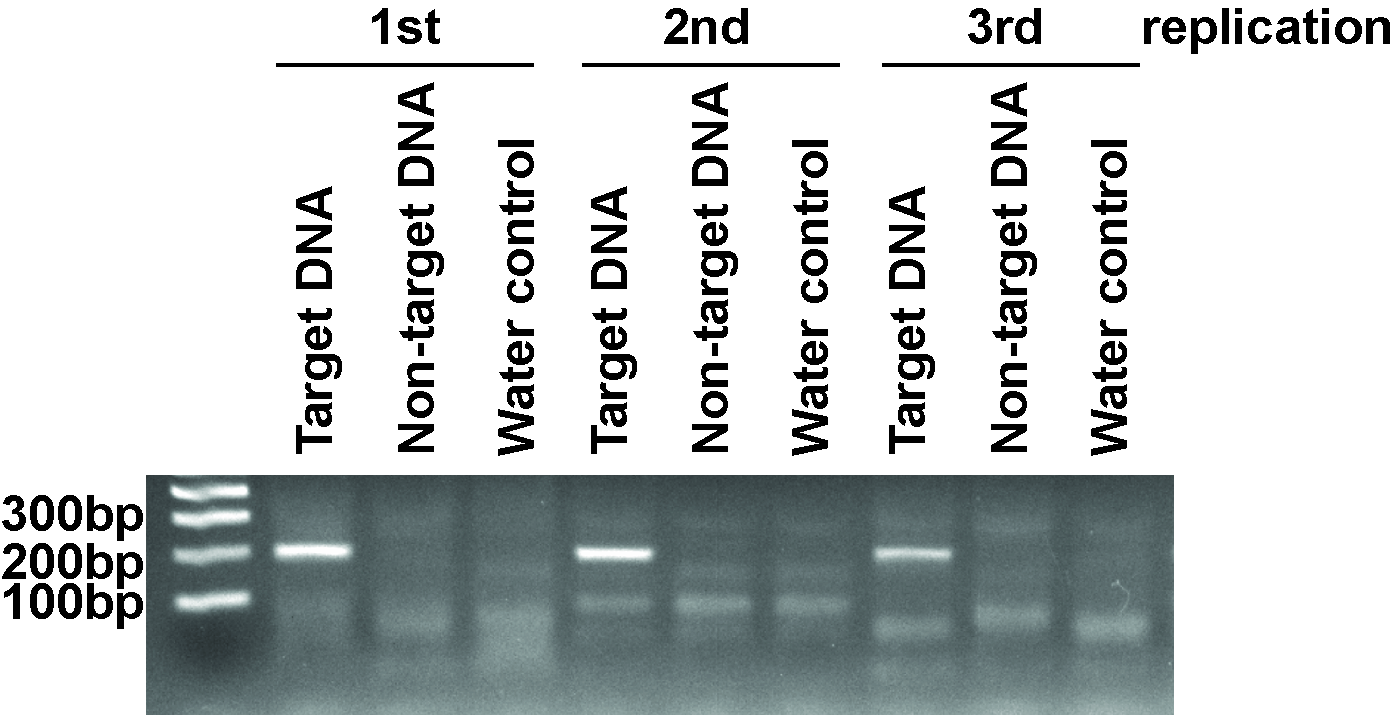

Supplement: S4 Fig — 1 ng F. oxysporum DNA was used as target DNA in reactions to produce 200 bp target amplicons. 1 ng A. thaliana DNA was used as non-target DNA. Reactions post amplification were analysed on agarose gel. (TIF) [file pone.0235216.s004.tif]

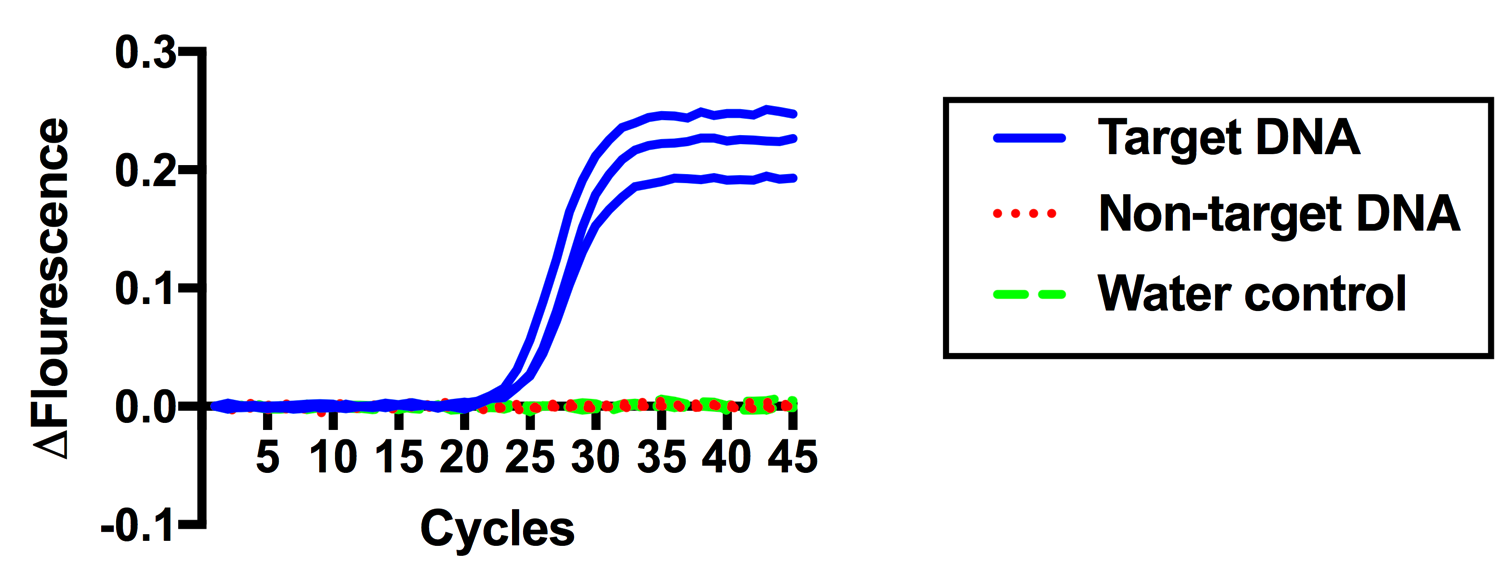

Supplement: S5 Fig — RPA primers were added into PCR reactions containing either 10 ng target DNA (blue solid line), non-target DNA (red dotted line) or water (green dotted line). (TIFF) [file pone.0235216.s005.tiff]

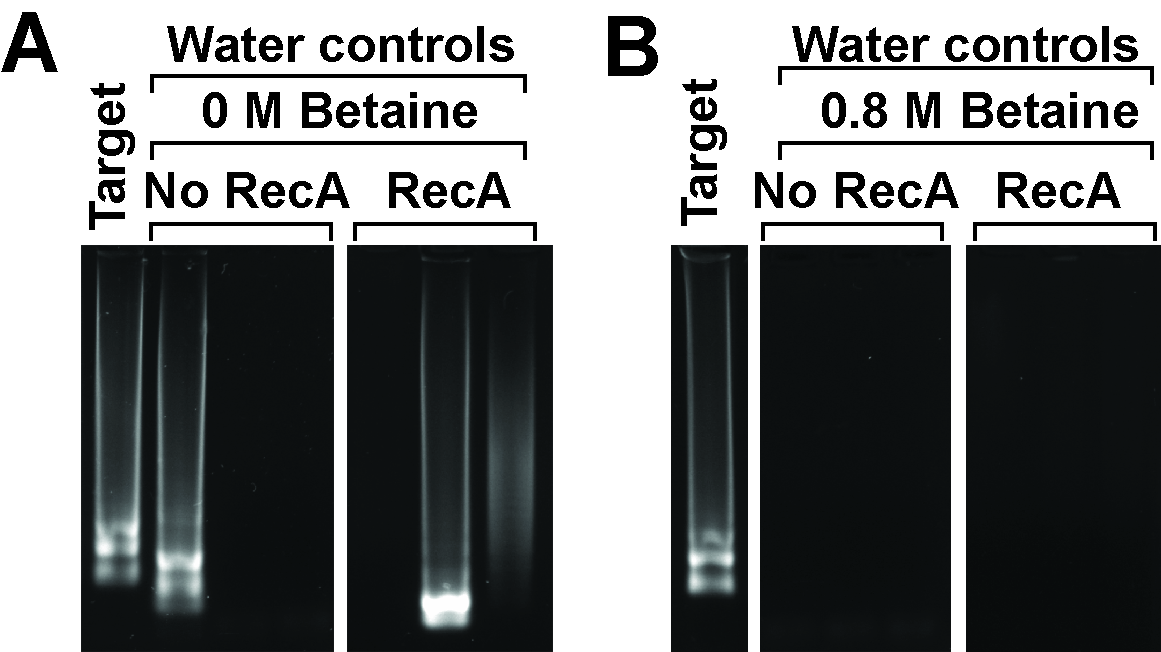

Supplement: S6 Fig — Water was added into reactions containing betaine and/or RecA. 1 ng F. oxysporum (target DNA) was added into the reaction without betaine and RecA as positive control. Images in each panel were generated from the same raw gel image, but re-arranged to remove irrelevant lanes in respective gel images (S1 Raw Images). (TIF) [file pone.0235216.s006.tif]
