# Supplementary material for: Evaluation and improvement of isothermal amplification methods for point-of-need plant disease diagnostics
Source: PLoS One. 2020 Jun 29;15(6):e0235216. doi: 10.1371/journal.pone.0235216 (PMC7323990; doi:10.1371/journal.pone.0235216)
Supplement: S1 Table — (DOCX) [file pone.0235216.s007.docx]

**S1 Table. Prominent primers used in this study**

| **Method** | **Primer names** | **Sequence (5’-3’)** |
| --- | --- | --- |
| LAMP | F3 | TTGCGAACGTCACTTACCAA |
|  | B3 | GAATATACCAATCTTGAGCAGAGCT |
|  | FIP | AGGGCCGCCGTTGAGATAGTCTTACCAACATCAGCAAGTATGG |
|  | BIP | GGAAAGCCCACCAACGGAGTTGTACCAGTGACCTTGATGAAC |
|  | Swarm forward primer | AGGGCCGCCGTTGAG |
|  | Swarm reverse primer | TGGAAAGCCCACCAACG |
|  | Loop forward primer | CTGCTGGACATCGAC |
|  | Loop reverse primer | AAGATCAGCGGCATCA |
| RPA | F | AACGTCACTTACCAAAACATTTCCCTTACCAAC |
|  | R | AGCTACCATCGCCACACAGAATATACCAATCTT |
| PSR | Ft | AGGGCCGCCGTTGAGATAGT-GTATTAGATTGCGAACGTC |
|  | Bt | TGATAGAGTTGCCGCCGGGAGAATATACCAATCTTGAGCAGAGCT |
|  | IF | CCATACTTGCTGATGTTGGTAAG |
|  | IB | GTTCATCAAGGTCACTGGT |
| CPA | 1s | GCCGCCGTTGAGATAGTCTTACCAACATCAGCAAGTATGG |
|  | 2a | TGTACCAGTGACCTTGATGAAC |
|  | 3a | CCTGCTGGACATCGACA |
|  | 4s | TTGCGAACGTCACTTACCAA |
|  | 5s | TGTACCAGTGACCTTGATGAAC |
|  | 6s | ACTCCGTTGGTGGGCTTTCC |
