# Supplementary material for: Evaluation and improvement of isothermal amplification methods for point-of-need plant disease diagnostics
Source: PLoS One. 2020 Jun 29;15(6):e0235216. doi: 10.1371/journal.pone.0235216 (PMC7323990; doi:10.1371/journal.pone.0235216)
Supplement: S1 Sequence — (DOCX) [file pone.0235216.s008.docx]

**S1 Sequence. 209 bp fragment from *F.conglutinans* (GenBank: AB256753.1) for primer design**

GTATTAGATTGCGAACGTCACTTACCAAAACATTTCCCTTACCAACATCAGCAAGTATGGTGTCGATGTCCAGCAGGACTATCTCAACGGCGGCCCTACTGGAAAGCCCACCAACGGAGTCAAGATCAGCGGCATCAAGTTCATCAAGGTCACTGGTACAGTGGCTAGCTCTGCTCAAGATTGGTATATTCTGTGTGGCGATGGTAGCT
