# Supplementary material for: Evaluation and improvement of isothermal amplification methods for point-of-need plant disease diagnostics
Source: PLoS One. 2020 Jun 29;15(6):e0235216. doi: 10.1371/journal.pone.0235216 (PMC7323990; doi:10.1371/journal.pone.0235216)

Original Gel Images

Title:

Evaluation and improvement of isothermal amplification for point-of-need plant disease diagnostics

Authors:

Yiping Zou, Michael Glenn Mason, Jose Ramon Botella

Author Affiliations:

School of Agriculture and Food Sciences, The University of Queensland, Brisbane, QLD, Australia

Corresponding authors:

Email: j.botella@uq.edu.au (JB) and michael.mason@uq.edu.au (MM)

Unless otherwise stated, original gel images were obtained by analysing amplification products on the 1% agarose gel containing 0.001% Ethidium Bromide Dye with the aid of BIO-RAD GelDoc. Original images used to generate final figures in main text and supporting information were included in this file and annotated using Adobe Illustrator with lanes not included in final figures labelled by “X”.

Fig 1

Panel (A) and (D)

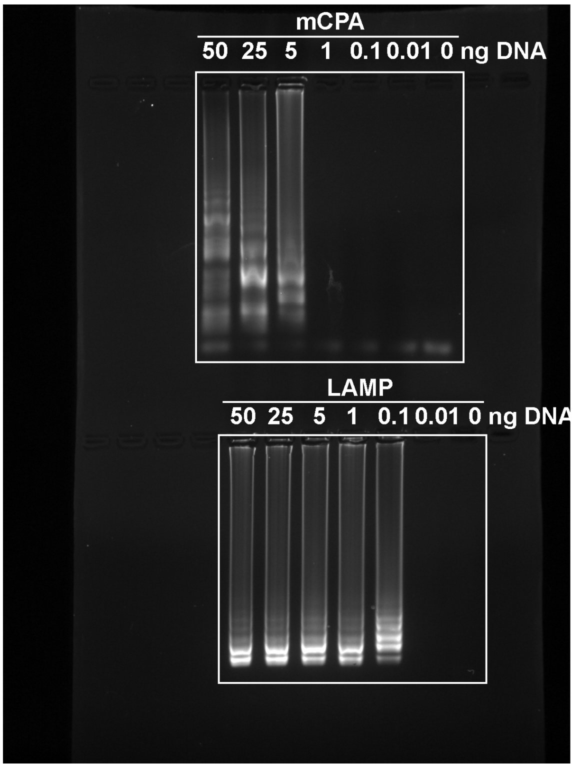

Panel (B) (RPA products were analysed on 2% agarose gel)

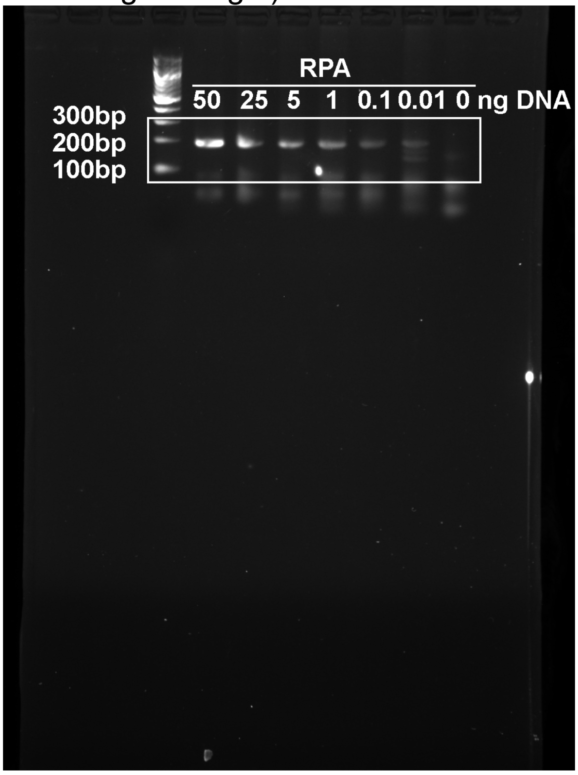

Panel (C)

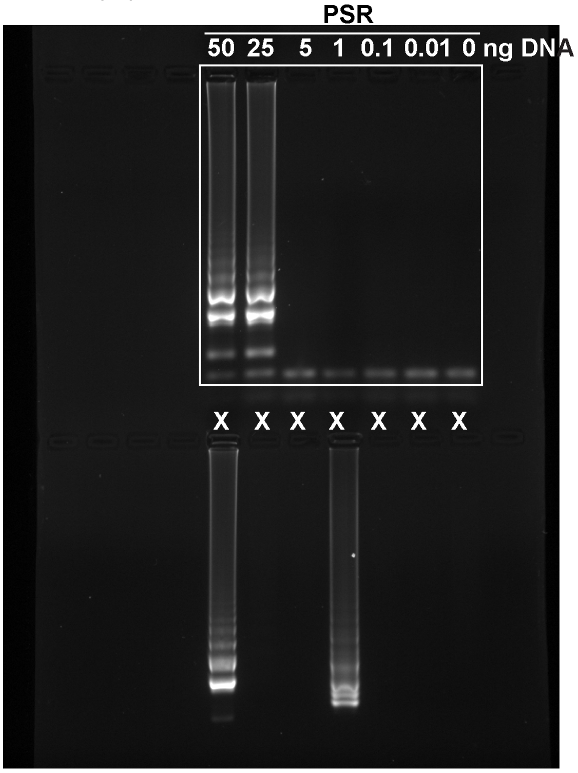

S2 Fig

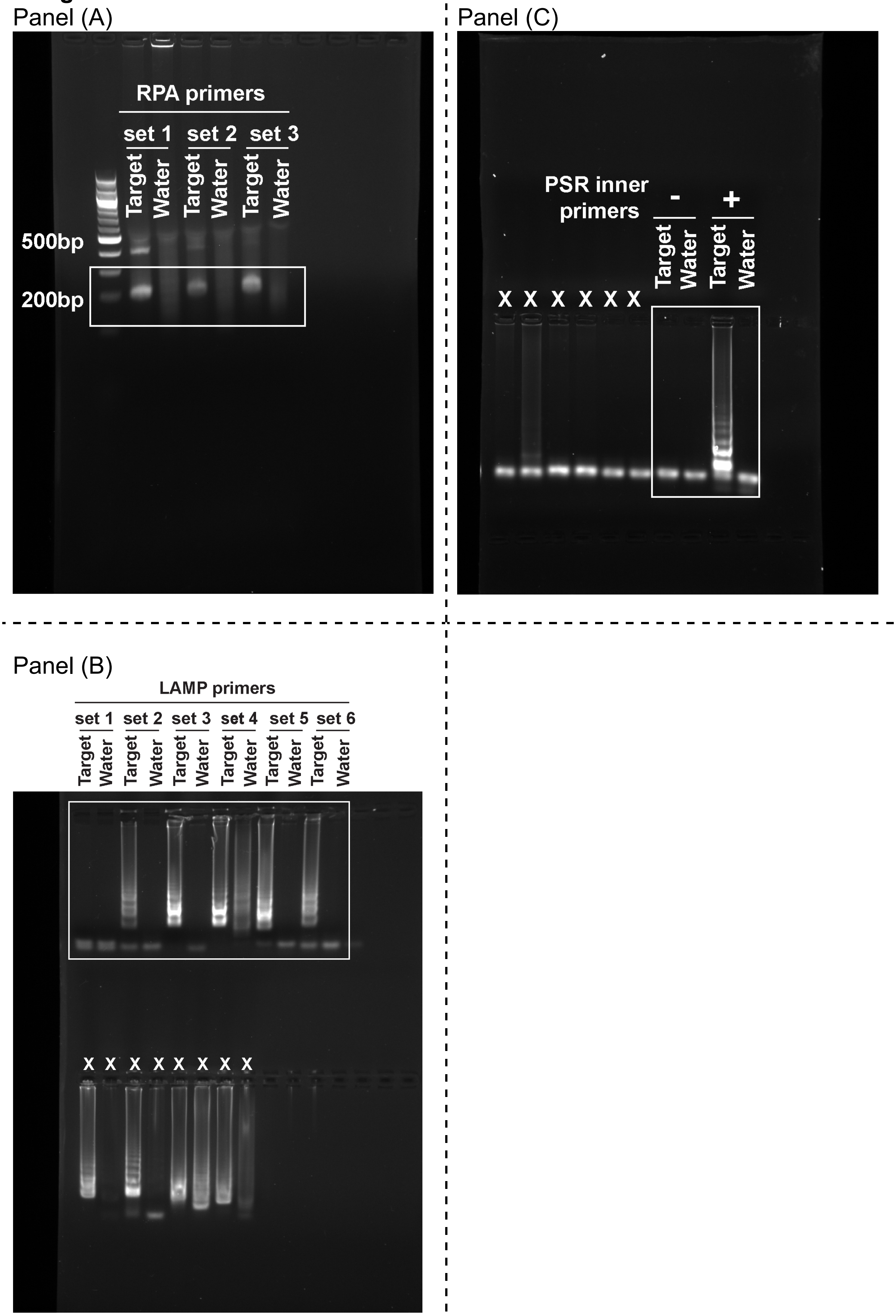
Panel (A)
RPA primers
set 1
set 2
set 3
Target
Water
Target
Water
Target
Water
500bp
200bp
Panel (C)
PSR inner primers
-
+
Target
Water
Target
Water
X X X X X X
Panel (B)
LAMP primers
set 1
set 2
set 3
set 4
set 5
set 6
Target
Water
Target
Water
Target
Water
Target
Water
Target
Water
Target
Water
X X X X X X X X

S3 Fig

Panel (A)

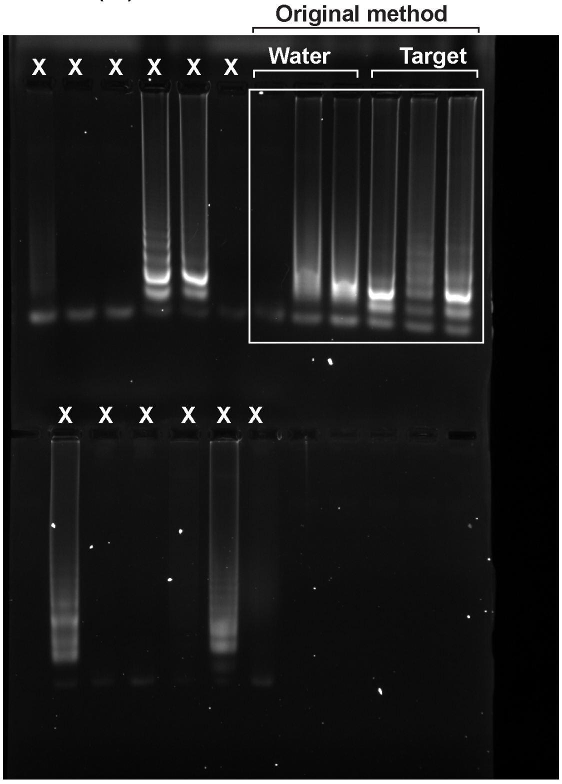

Panel (C)

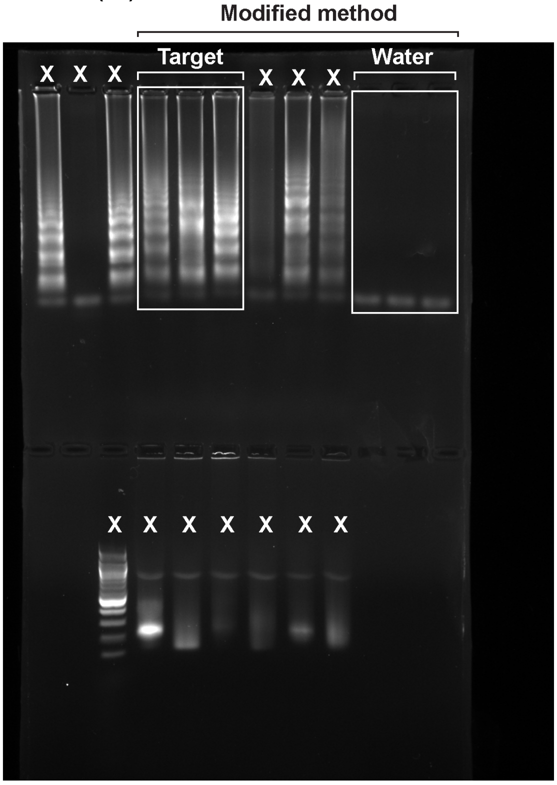

S4 Fig (Amplicons were analysed on 2% agarose gel)

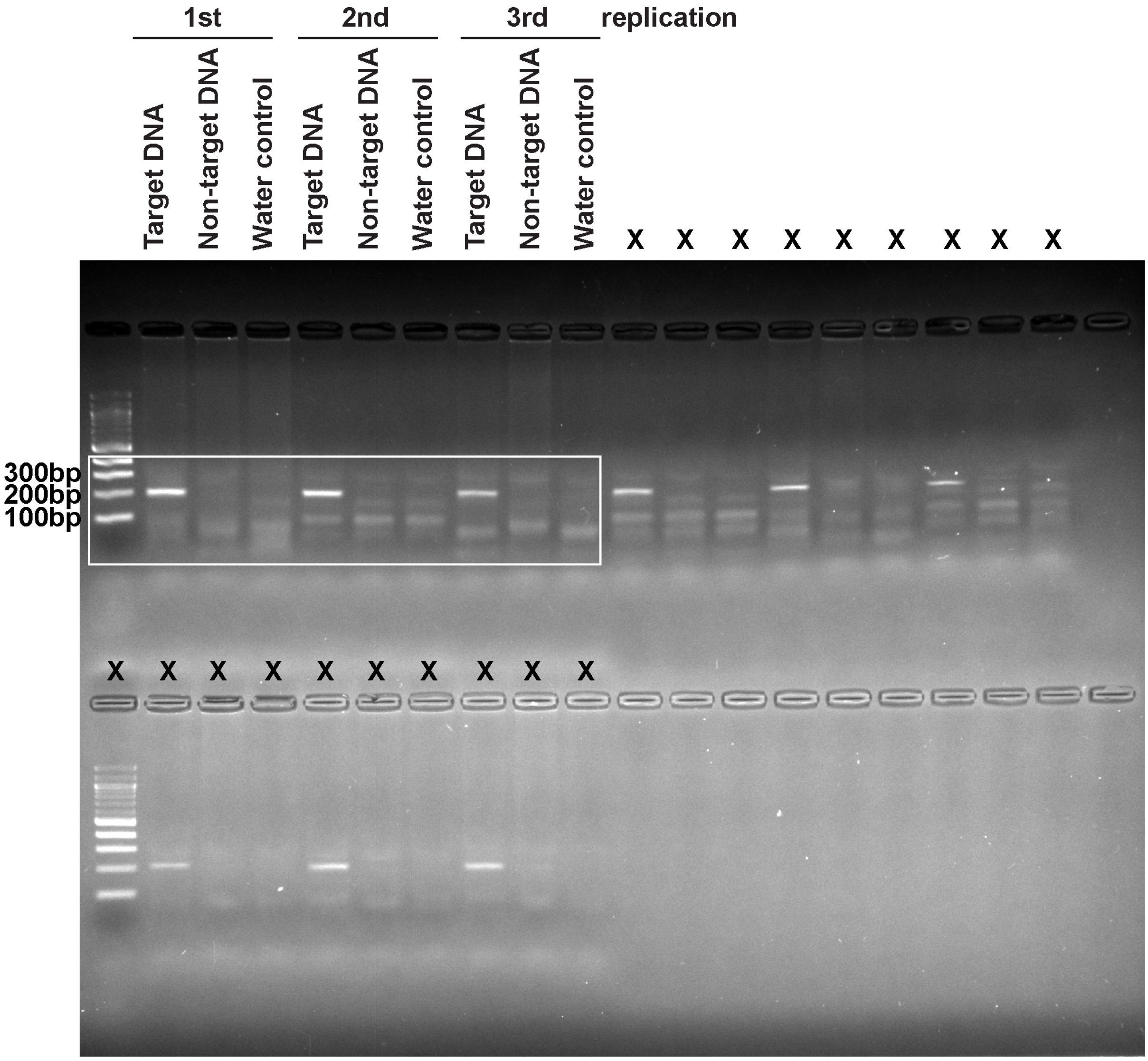

S6 Fig

Panel (A)

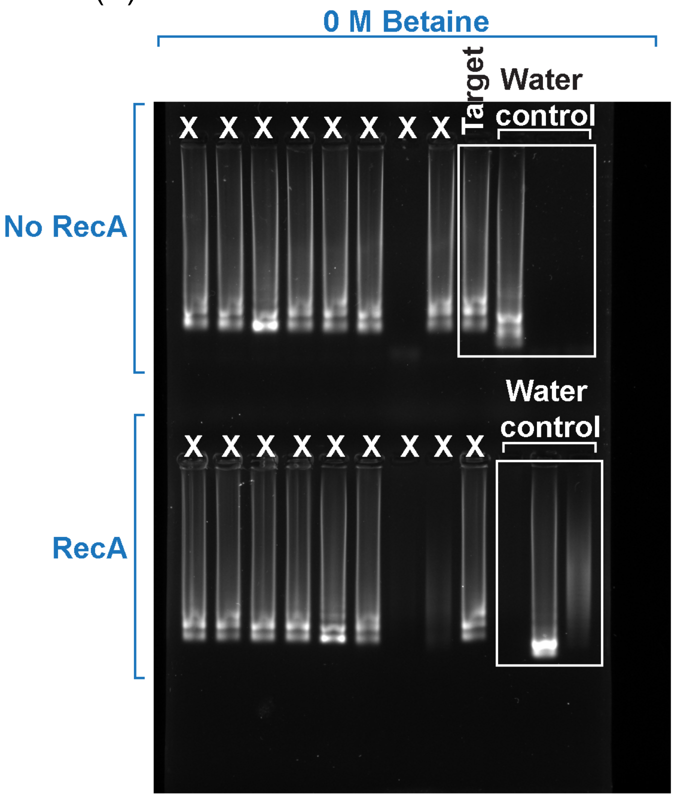

Panel (B)

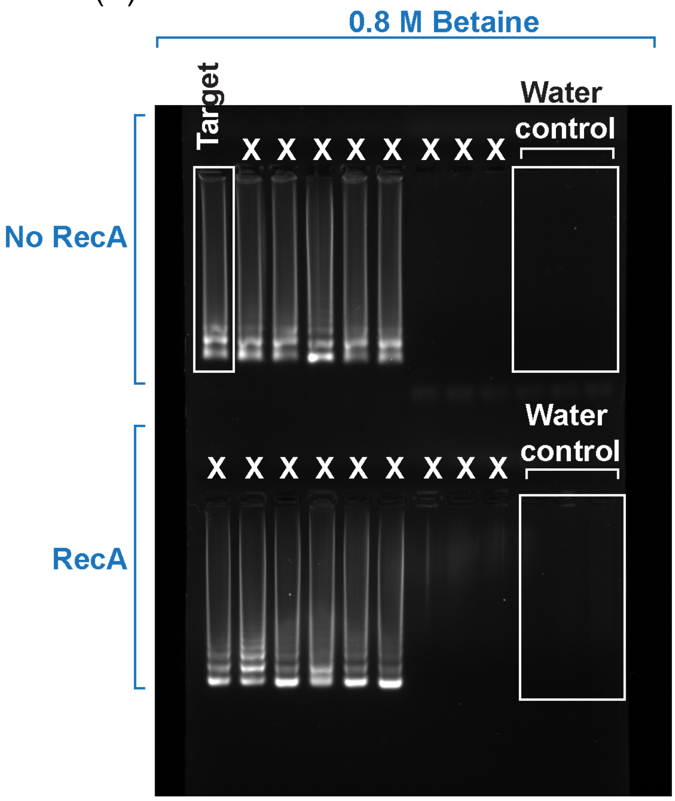

Supplement: S1 Raw Images — (PDF) [file pone.0235216.s010.pdf]
